# Burden of dementia attributable to smoking among adults aged ≥40 years: A secondary dataset analysis of Global Burden of Disease 1990−2021 with projections to 2035

**DOI:** 10.18332/tid/218789

**Published:** 2026-05-16

**Authors:** Xinhui Hu, Guihua Li, Kangle Dai, Sicheng Tang, Xiangyu Jin, Wenming Yang, Taotao Lu

**Affiliations:** 1 College of Rehabilitation Medicine, Anhui University of Chinese Medicine, Hefei City, China; 2 Department of Neurology, The First Affiliated Hospital of Anhui University of Chinese Medicine, Hefei City, China

**Keywords:** Alzheimer’s disease, DALYs, age-standardized rate, Global Burden of Disease study, projection

## Abstract

**INTRODUCTION:**

Dementia persists as a critical global health challenge. Smoking is a modifiable behavioral factor associated with dementia, although improvements in healthcare have reduced dementia prevalence and mortality. Evaluating long-term changes in the smoking-attributable dementia burden provides a useful reference for informing and contextualizing dementia prevention efforts.

**METHODS:**

This study is a secondary analysis of Global Burden of Disease (GBD) 2021 estimates. We examined smoking-attributable deaths and disability-adjusted life years (DALYs) for dementia among adults aged ≥40 years, across 204 countries and territories from 1990 to 2021. We assessed temporal trends using age-standardized rates and estimated annual percentage changes (EAPC), evaluated inequality across sociodemographic development levels, and projected the burden to 2035 using a Bayesian age–period–cohort (BAPC) model.

**RESULTS:**

In 2021, the global burden of dementia attributable to smoking reached 1533214 DALYs (95% UI: 635494–3540712), representing an approximate twofold increase compared with 1990. However, the age-standardized DALY rate (ASDR) declined significantly over the same period, with an EAPC of -0.88 (95% CI: -0.92 – -0.83). Population growth and population ageing were the dominant contributors to the increase in DALYs, accounting for 112.17% and 26.02%, respectively, whereas epidemiological improvements partially offset the burden (-38.19%). In terms of regional variation, East Asia bore the heaviest absolute burden. BAPC projections indicated that despite continued declines in ASDR, smoking-attributable dementia DALYs are expected to keep increasing through 2035.

**CONCLUSIONS:**

Despite declining age-standardized rates, the absolute burden of smoking-attributable dementia continues to rise, partly because reductions in smoking exposure are insufficient to counter demographic pressures from population ageing and growth. Persistent disparities across sociodemographic index groups further indicate that gains in tobacco control do not consistently translate into proportional reductions in dementia burden. Aligning tobacco control with ageing-responsive health system strategies will be essential to moderating future burden growth.

## INTRODUCTION

With the intensification of population ageing and lifestyle changes, Alzheimer’s disease (AD) and other dementias have become pressing global public health challenges. Among these, AD is the predominant type of dementia, accounting for approximately 60% to 80% of all cases^1^. As a progressive neurodegenerative disease, AD manifests initially with memory loss and cognitive decline, and ultimately impairs the capacity for daily functioning. The World Health Organization reported that dementia caused around 1.6 million deaths worldwide in 2019, ranking as the seventh leading cause of mortality globally. The total number of individuals living with dementia is expected to rise sharply to 139 million by 2050^2^, with the global macroeconomic burden of AD estimated to exceed $14 trillion^3^. The mechanisms underlying AD onset and progression remain incompletely understood, and current treatments are limited to symptomatic management^4^. Epidemiological evidence suggests that dementia is associated with several modifiable behavioral risk factors, including tobacco use, alcohol consumption, unhealthy diet, and physical inactivity^5^. Therefore, focusing on modifiable risk factors and implementing effective preventive strategies is considered a key approach to reducing the burden of dementia and slowing its progression.

As a leading global health risk factor, smoking is strongly linked to numerous chronic conditions, such as cardiovascular and respiratory diseases^6-8^. Official collaborators have reported evidence that smoking ranks third globally in terms of risk factors for disease burden across all age categories, following particulate matter air pollution and high systolic blood pressure^9^. The 2024 updated report from the Lancet Commission on Dementia Prevention, Intervention, and Care indicates that reducing smoking may help lower the incidence of age-related dementia^10^. Toxic components in tobacco smoke exacerbate AD pathology through oxidative stress and inflammatory responses, thereby promoting abnormal amyloid-β plaque deposition and tau protein hyperphosphorylation^11^. Compared with non-smokers, long-term smokers exhibited marked reductions in total and subregional hippocampal volumes bilaterally, as revealed by MRI. Moreover, healthy older adults with a history of smoking showed greater regional brain atrophy over a two-year period than non-smokers^12,13^, resembling neuropathological changes characteristic of AD. A European survey found that smoking substantially modified the relationship between lifestyle and cognitive impairment: smokers experienced faster cognitive decline, whereas non-smokers showed similar rates of change^14^. Consistently, according to a Japanese cohort study, individuals who had quit smoking for at least three years exhibited a risk of developing dementia that was equivalent to that of never smokers^15^.

Given the long latency between midlife smoking exposure and dementia onset, the consequences of current smoking patterns will unfold over future decades, highlighting the need for long-term assessment and projections of dementia burden. Currently, available studies on the long-term impact of smoking on dementia have focused on specific regions or populations, and few have incorporated future projections^16-18^. By providing a comprehensive and comparable assessment based on the Global Burden of Disease (GBD) 2021 estimates across regions and demographic groups, this study aims to describe and project the burden of dementia attributable to smoking.

## METHODS

### Data sources

Using the GBD 2021 estimates, we conducted a secondary analysis of smoking-attributable burdens of AD and other dementias from 1990 to 2021 among adults aged ≥40 years across 204 countries and territories. Based on the study aim, the included data comprised smoking-attributable deaths and disability-adjusted life years (DALYs) for dementia at the global, regional, and national levels. The Global Health Data Exchange website provided publicly accessible data, which were accessed in April 2025; data on dementia burden attributable to smoking were extracted for subsequent analyses^19^.

### Disease definition

In this study, dementia was defined as the GBD 2021 cause category ‘Alzheimer’s disease and other dementias’ as classified on the Global Health Data Exchange platform, representing an all-cause dementia outcome that covers all dementia subtypes. Dementia cases were identified using International Classification of Diseases (ICD) codes, including ICD-10 codes F00–F03.9, G30–G31.1, and G31.8–G31.9, as well as ICD-9 codes 290–290.9, 294.1–294.9, and 331–331.2^20^. Smoking refers to current daily (every day) or occasional (non-daily, less than daily) use of smoked tobacco products. Smoked tobacco products were defined as tobacco products consumed by smoking and did not include smokeless tobacco, heated tobacco products, chewing tobacco, cannabis, or e-cigarettes.

### Statistical analysis

To minimize heterogeneity arising from differences in population age structure, age-standardized rates (ASRs) were derived through direct standardization, applying the global age distribution as the reference. Stratified analyses were prespecified by sex (male, female), by age in 5-year groups (40–44 to ≥95 years), and by sociodemographic index (SDI) quintiles (low to high), to characterize heterogeneity in dementia burden attributable to smoking across demographic groups and levels of development. Data on deaths and DALYs attributable to smoking-related dementia from 1990 to 2021 were collected at the global, national, and regional levels.

A Bayesian framework was applied to generate posterior distributions for each parameter in order to account for the uncertainty inherent in point estimates. Final estimates were obtained as the average of 500 draws, with 95% uncertainty intervals (UIs) determined by the 2.5th and 97.5th percentiles^9^. Long-term trends in age-standardized mortality rate (ASMR) and age-standardized DALY rate (ASDR) due to smoking-related dementia were evaluated by estimating the annual percentage change (EAPC) through linear regression. ASR was deemed increasing when both the EAPC and its 95% CI lower bound exceeded 0, decreasing when both were below 0, and stable otherwise.

To comprehensively assess disparities, drivers, and future trends in smoking-attributable dementia burden, the analysis incorporated several complementary analytical approaches. Frontier analysis was used to evaluate the relative efficiency of countries and territories in achieving health outcomes in relation to SDI. Health inequality was assessed using the slope index of inequality (SII) and the concentration index to quantify absolute and relative socio-economic disparities. In addition, the Das Gupta decomposition method was employed to disentangle the contributions of population ageing, population growth, and epidemiological changes to trends in deaths and DALYs. Future burden through 2035 was projected using a Bayesian age–period–cohort (BAPC) model based on data from 1990 to 2021. Detailed descriptions of these analytical methods are provided in the Supplementary file.

Statistical analyses and data visualizations were performed in R software (version 4.4.2)^21^ and the JD_GBDR package (version 2.7.2, Jingding Medical Technology Co., Ltd.)^22^. The means of descriptive statistics were displayed with either 95% UI or 95% CI. It was deemed statistically significant when the two-sided p-value was <0.05.

## RESULTS

### Global burden of AD and other dementias attributable to smoking in 2021

At the global level, deaths from AD and other dementias attributable to smoking increased from 32165 (95% UI: 7167–90875) in 1990 to 67176 (95% UI: 15334-188054) in 2021, representing an approximate twofold rise. Similarly, DALYs increased from 794915 (95% UI: 328160-1832306) to 1533214 (95% UI: 635494–3540712) over the same period, showing a comparable upward trend (Supplementary file Table S1). In 2021, the ASMR and ASDR were 2.50 (95% UI: 0.57-6.98) and 54.84 (95% UI: 22.67–126.54) per 100000 population, respectively, both markedly higher in males than in females. Between 1990 and 2021, ASMR (EAPC: -0.95; 95% CI: -0.99 – -0.91) and ASDR (EAPC: -0.88; 95% CI: -0.92 – -0.83) attributable to smoking exhibited a significant downward trend (Table 1).

**Table 1:** Global and regional age-standardised mortality rate (ASMR), age-standardised disability-adjusted life year rate (ASDR), and estimated annual percentage change (EAPC) for Alzheimer’s disease and other dementias attributable to smoking, 1990 to 2021, across five sociodemographic index regions and 21 GBD regions, based on Global Burden of Disease (GBD) 2021 estimates

	*Age-standardised rate per 100000 people (95% UI)*				*Estimated annual percentage change from 1990 to 2021 (95% CI)*	
	*1990*		*2021*			
	*Death rate*	*DALY rate*	*Death rate*	*DALY rate*	*Death rate*	*DALY rate*
**Global**	3.24 (0.73–9.04)	69.69 (28.50–160.45)	2.50 (0.57–6.98)	54.84 (22.67–126.54)	-0.95 (-0.99 – -0.91)	-0.88 (-0.92 – -0.83)
**Sex**						
Male	5.32 (1.19–15.26)	111.87 (45.32–262.65)	4.28 (0.95–12.25)	91.30(37.18–21 4.97)	-0.79 (-0.86 – -0.73)	-0.73 (-0.80 – -0.66)
Female	1.99 (0.46–5.38)	40.78 (16.78–91.42)	1.29 (0.30–3.41)	26.90 (11.13–59.68)	-1.60 (-1.68 – -1.52)	-1.50 (-1.56 – -1.45)
**Sociodemographic index region**						
Low	1.40 (0.30–4.09)	29.03 (11.75–68.84)	0.41 (0.10–1.18)	24.20 (9.31–58.88)	-0.46 (-0.54 – -0.39)	-0.64 (-0.70 – -0.59)
Low-middle	2.23 (0.50–6.59)	47.65 (19.52–113.96)	0.63 (0.14–1.81)	38.90 (15.28–91.55)	-0.55 (-0.59 – -0.51)	-0.70 (-0.72 – -0.68)
Middle	3.32 (0.73–9.34)	72.11 (28.88–166.65)	0.88 (0.21–2.45)	57.88 (23.63–136.70)	-0.95 (-1.01 – -0.89)	-0.88 (-0.94 – -0.81)
High-middle	3.08 (0.68–8.56)	67.86 (27.14–156.53)	0.96 (0.22–2.60)	65.61 (26.88–151.62)	-0.31 (-0.33 – -0.28)	-0.17 (-0.19 – -0.14)
High	3.77 (0.86–10.42)	83.55 (34.96–189.12)	0.8 5(0.20–2.36)	56.92 (23.73–127.85)	-1.41 (-1.47 – -1.35)	-1.34 (-1.40 – -1.28)
**GBD region**						
Andean Latin America	0.96 (0.20–2.82)	21.06 (8.40–49.91)	0.77 (0.16–2.21)	16.88 (6.76–39.40)	-0.85 (-0.96 – -0.74)	-0.84 (-0.93 – -0.74)
Australasia	2.31 (0.52–6.48)	56.34 (23.53–127.32)	1.61 (0.37–4.50)	36.17 (14.55–82.30)	-1.10 (-1.19 – -1.01)	-1.40 (-1.47 – -1.34)
Caribbean	1.81 (0.38–5.29)	43.30 (18.00–99.09)	1.38 (0.30–3.98)	33.10 (13.87–75.36)	-1.05 (-1.19 – -0.91)	-1.06 (-1.20 – -0.93)
Central Asia	1.24 (0.27–3.61)	30.34 (12.55–68.87)	1.46 (0.32–4.29)	34.21 (14.15–79.77)	0.96 (0.82–1.10)	0.74 (0.59–0.88)
Central Europe	2.26 (0.48–6.51)	56.98 (23.62–129.49)	1.68 (0.36–4.75)	44.13 (18.62–99.40)	-0.99 (-1.08 – -0.90)	-0.84 (-0.90 – -0.78)
Central Latin America	1.65 (0.36–4.79)	38.51 (16.47–87.14)	0.84 (0.19–2.39)	20.15 (8.39–45.47)	-2.36 (-2.48 – -2.23)	-2.25 (-2.37 – -2.13)
Central Sub-Saharan Africa	0.79 (0.17–2.43)	17.58 (6.74–44.45)	0.67 (0.14–1.93)	15.08 (5.46–36.66)	-0.51 (-0.81 – -0.21)	-0.39 (-0.67 – -0.10)
East Asia	4.98 (1.06–13.69)	100.60 (37.96–234.07)	4.12 (0.90–11.99)	89.46 (35.80–210.60)	-0.73 (-0.81 – -0.65)	-0.48 (-0.58 – -0.39)
Eastern Europe	1.26 (0.27–3.65)	32.30 (13.35–73.87)	1.35 (0.29–3.93)	35.60 (14.48–81.56)	0.39 (0.12–0.67)	0.43 (0.15–0.71)
Eastern Sub-Saharan Africa	1.45 (0.32–4.15)	28.49 (11.22–68.41)	1.03 (0.23–2.91)	20.63 (7.84–49.45)	-1.25 (-1.35 – -1.15)	-1.17 (-1.27 – -1.07)
High-income Asia Pacific	3.56 (0.80–10.09)	74.22 (29.45–173.59)	2.15 (0.49–5.82)	47.23 (19.21–105.07)	-1.77 (-1.85 – -1.70)	-1.57 (-1.62 – -1.51)
High-income North America	4.41 (0.99–12.20)	103.00 (43.30–234.03)	3.14 (0.71–8.61)	69.66 (28.87–157.50)	-1.25 (-1.32 – -1.18)	-1.40 (-1.47 – -1.33)
North Africa and Middle East	3.34 (0.74–9.44)	71.30 (29.20–163.19)	2.55 (0.57–7.23)	54.42 (22.29–125.99)	-0.97 (-1.06 – -0.88)	-0.96 (-1.02 – -0.89)
Oceania	1.52 (0.32–4.38)	38.17 (14.88–90.17)	1.21 (0.26–3.53)	31.45 (12.33–74.40)	-0.86 (-0.93 – -0.79)	-0.73 (-0.79 – -0.67)
South Asia	2.00 (0.43–6.08)	41.64 (16.81–101.01)	1.66 (0.36–4.86)	32.71 (12.41–78.03)	-0.60 (-0.68 – -0.53)	-0.84 (-0.88 – -0.81)
Southeast Asia	2.77 (0.61–7.89)	62.08 (26.09–141.13)	2.23 (0.50–6.34)	48.31 (19.56–113.68)	-0.94 (-1.09 – -0.80)	-0.99 (-1.10 – -0.89)
Southern Latin America	1.33 (0.28–3.83)	37.62 (15.48–84.38)	1.15 (0.24–3.33)	31.45 (12.71–71.36)	-0.38 (-0.50 – -0.26)	-0.56 (-0.67 – -0.45)
Southern Sub-Saharan Africa	2.62 (0.58–7.50)	55.57 (22.46–127.96)	1.12 (0.23–3.28)	25.19 (9.62–60.24)	-2.90 (-2.99 – -2.80)	-2.65 (-2.74 – -2.56)
Tropical Latin America	4.81 (1.07–13.61)	104.26 (40.75–242.80)	2.73 (0.63–7.63)	62.16 (24.82–143.55)	-2.03 (-2.22 – -1.85)	-1.87 (-2.06 – -1.68)
Western Europe	3.61 (0.82–9.97)	77.02 (32.09–174.93)	2.33 (0.54–6.27)	51.48 (21.46–114.56)	-1.49 (-1.53 – -1.45)	-1.34 (-1.38 – -1.31)
Western Sub-Saharan Africa	0.55 (0.12–1.56)	11.10 (4.24–26.38)	0.42 (0.09–1.26)	8.82 (3.26–21.93)	-0.82 (-0.86 – -0.77)	-0.74 (-0.76 – -0.72)

UI: uncertainty interval. CI: confidence interval.

At the SDI regional level, both deaths and DALYs due to smoking-related dementia declined across all regions. The region with the highest burden shifted from the high SDI group in 1990 to the high-middle SDI group in 2021 (Supplementary file Table S1). All five SDI regions revealed overall decreases in ASMR and ASDR. However, the middle and high-middle SDI regions experienced an upward reversal in 2020, likely reflecting the impact of the COVID-19 pandemic on dementia-related outcomes. In 2021, the high-middle SDI region had the highest ASMR (0.96; 95% UI: 0.22-2.60) and ASDR (65.61; 95% UI: 26.88–151.62), with the least evident decline over time. Conversely, the high SDI region achieved the sharpest improvements, with EAPCs of -1.41 (95% CI: -1.47 – -1.35) for ASMR and -1.34 (95% CI: -1.40 – -1.28) for ASDR (Table 1; and Supplementary file Figures S1A and S1B).

At the level of the 21 GBD regions, the dementia burden attributable to smoking showed considerable geographical variation. In 2021, the highest ASDR was observed in East Asia, reaching 89.46 per 100000 population (95% UI: 35.80-210.60), followed by high-income North America (69.66; 95% UI: 28.87–157.50). In contrast, the lowest ASDR was recorded in Western Sub-Saharan Africa, at just 8.82 per 100000 (95% UI: 3.26–21.93). Over the past three decades, Southern Sub-Saharan Africa experienced the steepest declines globally in both ASMR (EAPC= -2.90; 95% UI: -2.99 – -2.80) and ASDR (EAPC= -2.65; 95% UI: -2.74 – -2.56). In comparison, Central Asia and Eastern Europe were the only regions where both ASMR and ASDR displayed increasing trends. In Central Asia, the EAPC for ASMR was 0.96 (95% UI: 0.82-1.10) and for ASDR was 0.74 (95% UI: 0.59–0.88), while in Eastern Europe, the respective EAPCs were 0.39 (95% UI: 0.12–0.67) and 0.43 (95% UI: 0.15-0.71) (Table 1; and Supplementary file Figures S1C–S1F).

**Figure 1:**
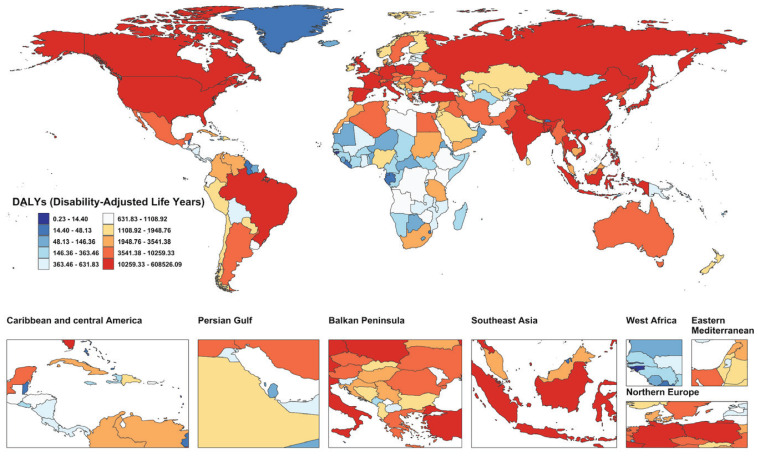
Global map of smoking-attributable disability-adjusted life years (DALYs) for Alzheimer’s disease and other dementias in 204 countries and territories in 2021, based on Global Burden of Disease (GBD) 2021 estimates

**Figure 2:**
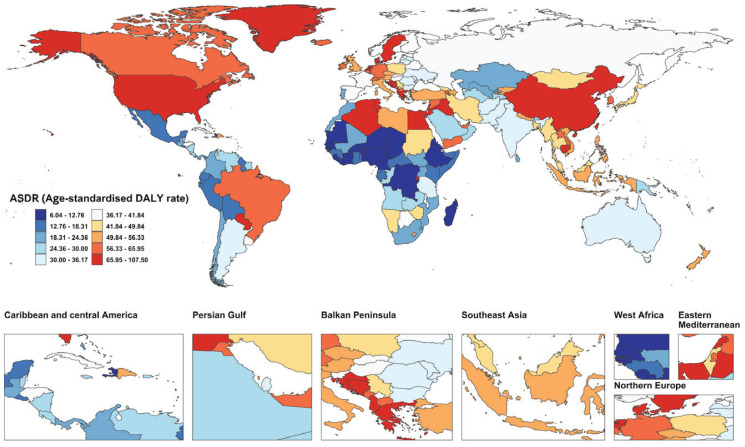
Global map of smoking-attributable age-standardized disability-adjusted life year rate (ASDR) for Alzheimer’s disease and other dementias in 204 countries and territories in 2021, based on Global Burden of Disease (GBD) 2021 estimates

**Figure 3:**
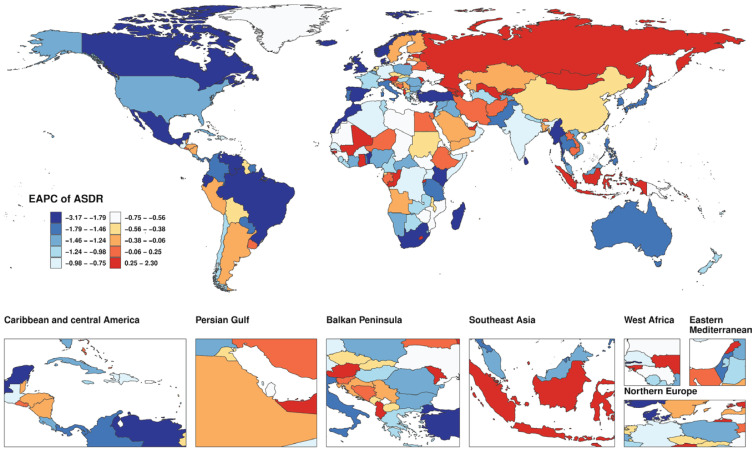
Global map of estimated annual percentage change (EAPC) in smoking-attributable age-standardized disability-adjusted life year rate (ASDR) for Alzheimer’s disease and other dementias in 204 countries and territories from 1990 to 2021, based on Global Burden of Disease (GBD) 2021 estimates

Among the 204 countries and territories, China, the United States, India, and Japan reported the highest numbers of smoking-attributable deaths and DALYs related to AD and other dementias in 2021 (Figure 1; and Supplementary file Figure S2A and Table S2). Lebanon reported the highest ASMR and ASDR, with China ranking second in ASDR. The greatest reductions in ASDR were recorded in South Africa, Mexico, Sri Lanka, Madagascar, Ireland, and Norway, with corresponding EAPCs of -3.14, -3.14, -2.84, -2.82, -2.70, and -2.70. Notably, these six countries exhibited an identical ranking in terms of ASMR decline, reflecting consistent downward trends (Figures 2 and 3; and Supplementary file Figures S2B and S2C and Table S2).

### Global burden of AD and other dementias attributable to smoking by sex and age

**Figure 4:**
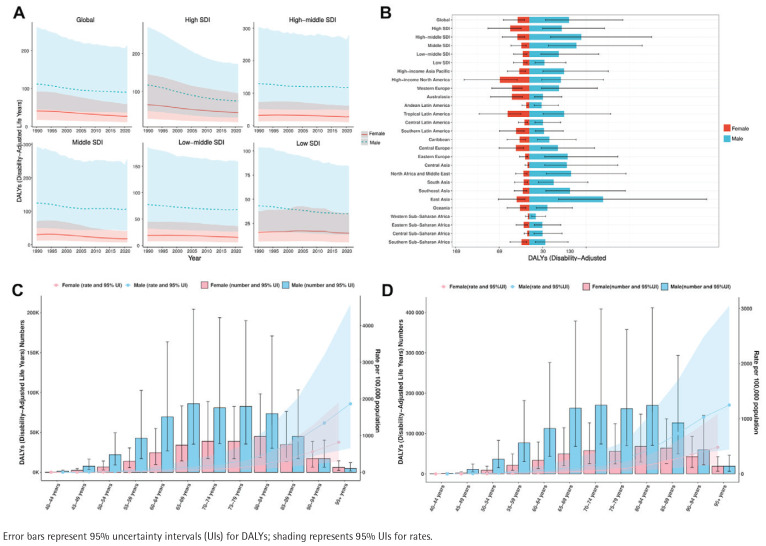
Temporal trends and distributions of disability-adjusted life years (DALYs) for dementia attributable to smoking, by region, age, and sex, based on the Global Burden of Disease (GBD) 2021 study: A) Temporal trend of age-standardized disability-adjusted life year rate (ASDR) from 1990 to 2021 at the global level and across 21 GBD regions; B) Distribution of ASDR by sex across 21 GBD regions in 2021; C) DALYs and ASDR in 1990 by age and sex; D) DALYs and ASDR in 2021 by age and sex

In 2021, there were 20420 deaths (95% UI: 4810–54048) from smoking-attributable AD and other dementias among females globally, compared to 46755 deaths (95% UI: 10329–134861) among males. Males also exhibited higher numbers of DALYs, as well as higher ASMR and ASDR values, than females. Although the global number of smoking-related deaths and DALYs increased for both sexes, ASMR and ASDR showed overall declining trends. Specifically, ASMR decreased with an EAPC of -0.79 (95% CI: -0.86 – -0.73) in males and -1.60 (95% CI: -1.68 – -1.52) in females, while ASDR dropped with an EAPC of -0.73 (95% CI: -0.80 – -0.66) in males and -1.50 (95% CI: -1.56 – -1.45) in females (Table 1; and Supplementary file Table S1). The declining trend was more pronounced in females, but the absolute burden remained consistently higher in males. Within the five SDI regions, the most substantial reductions in both ASDR and ASMR occurred in high SDI regions. Regarding ASDR, improvements were most evident among females in high SDI regions, while females in low SDI regions showed the smallest reductions. For ASMR, the decline was most striking in males from high-SDI regions, whereas the smallest decrease in mortality was observed in females from low-SDI regions (Figure 4A; and Supplementary file Figure S3A). Across the 21 GBD regions, females had the highest ASMR and ASDR in high-income North America, whereas males in East Asia exhibited the highest values for both measures (Figure 4B; and Supplementary file Figure S3B).

Figures 4C and 4D, and Supplementary file Figures S3C and S3D, illustrate deaths, DALYs, and their corresponding age-specific rates for smoking-related dementia across global age groups in 1990 and 2021. The peak number of deaths for both males and females occurred in the 80-84 years age group in 1990. By 2021, this peak remained unchanged for males but moved to the 85-89 years age group in females. In contrast, in 2021, DALYs in females peaked at ages 80-84 years, while in males the peak shifted from ages 65-69 years in 1990 to 70-74 years in 2021. Additionally, in 2021, deaths among males exceeded those among females in all age groups below 95 years. In the ≥95 years age group, female deaths surpassed those of males. DALYs followed the same trend. Among all age groups aged ≥40 years, male ASMR and ASDR values consistently exceeded those in females.

### Association of SDI with smoking-attributable burden of AD and other dementias

Considerable variation in the burden of dementia due to smoking was observed across regions with different SDI levels. As illustrated in Supplementary file Figure S4A, analysis of 21 GBD regions from 1990 to 2021 revealed a nonlinear ‘N-shaped’ association between ASDR and SDI: ASDR rose with SDI values below 0.55, declined when SDI ranged from roughly 0.55 to 0.65, and increased again once SDI exceeded 0.65. An analogous relationship was detected between ASMR and SDI (Supplementary file Figure S4B). The highest burden attributable to smoking-related dementia was found in East Asia, a high-middle SDI region. Spearman’s rank test indicated a moderate positive correlation between ASDR and SDI (r=0.4665, p<0.001), with a similar relationship for ASMR (r=0.4138, p<0.001). Compared with the other three SDI regions, both ASDR and ASMR were notably lower in low and low-middle SDI regions (Table 1).

In the Spearman rank correlation analysis across 204 countries and territories, both ASDR and ASMR remained positively associated with SDI overall (r=0.4533, p<0.001; r=0.3968, p<0.001). However, substantial regional heterogeneity and nonlinear variation patterns were also evident. Notably, certain countries, such as Lebanon, exhibited disproportionately high ASDR and ASMR values relative to others with similar SDI levels, further highlighting disparities in national tobacco control policies. EAPC demonstrated weak positive associations with both ASDR and ASMR in 2021. Conversely, the EAPCs of ASDR (r= -0.078, p=0.27) and ASMR (r= -0.11, p=0.11) showed negative correlations with SDI (Supplementary file Figures S4C–S4F).

### Frontier analysis of ASDR attributable to smoking-related AD and other dementias

Frontier analysis based on ASDR and SDI (1990–2021) was conducted across 204 countries and territories to evaluate the scope for reducing the dementia burden attributable to smoking (Supplementary file Figures S5A and S5B and Table S3). Among the 204 countries and territories assessed, the 15 with the widest gaps between actual and optimal performance were Lebanon, China, Albania, Jordan, Kiribati, Egypt, Greenland, Denmark, Rwanda, Cambodia, Algeria, Paraguay, Iraq, Tunisia, and Greece (effective difference range: 100.73–66.72). These countries were farthest from the frontier line, indicating substantial potential to improve performance in managing the burden of smoking-related dementia. High-performing frontier countries within the low SDI group included Somalia, Burkina Faso, Niger, Benin, and Guinea-Bissau. In comparison, several high SDI countries and territories, including Denmark, the Netherlands, the United States, Sweden, and the Republic of Korea, were farthest from the expected performance level. Despite their relatively high levels of socio-economic development, these countries achieved outcomes that were not entirely satisfactory in reducing the burden of smoking-related dementia.

### Health inequalities in smoking-attributable dementia burden by SDI

Between 1990 and 2021, both absolute and relative inequalities in smoking-attributable dementia burden declined. In 1990, the SII for DALYs was 35.19 per 100000 population (95% CI: 24.92–45.46), decreasing to 25.70 in 2021 (95% CI: 18.22–33.18). Supplementary file Figure S5C illustrates that ASDRs were consistently higher in countries with higher SDI levels, although the extent of inequality gradually declined over time. The concentration index decreased slightly from 0.14 (95% CI: 0.10–0.18) in 1990 to 0.13 (95% CI: 0.09–0.16) in 2021 (Supplementary file Figure S5D), indicating that the concentration of smoking-related dementia burden in high-SDI countries has diminished modestly. While some regions have experienced a narrowing of socio-economic disparities, global inequality in the distribution of smoking-attributable dementia continues to pose a crucial public health challenge.

### Decomposition analysis of changes in the smoking-attributable burden of AD and other dementias

Decomposition analysis quantified the contributions of population ageing, population growth, and epidemiological changes to trends in smoking-attributable dementia DALYs from 1990 to 2021. As presented in Supplementary file Figure S6, both population growth (112.17%) and ageing (26.02%) were the major drivers of the global increase, whereas epidemiological improvements partly offset this burden (-38.19%). In high SDI regions, ageing contributed the most to the increase in smoking-related dementia DALYs (71.98%), while population growth also played a major role (142.85%), and improvements in epidemiological profiles substantially reduced the burden (-114.83%). Across the other SDI levels, the contribution of population growth varied, accounting for 128.16%, 109.93%, 101.82%, and 81.03% in low, low-middle, middle, and high-middle SDI regions, respectively.

### Future trend predictions for smoking-attributable burden of AD and other dementias

Between 1990 and 2035, DALYs and deaths due to AD and other dementias attributable to smoking are expected to continue rising (Supplementary file Figures S7A and S8A). Globally, the number of deaths increased from approximately 32165 in 1990 to 67176 in 2021 and is projected to exceed 86337 by 2035, reflecting a steady upward trend.

At the global level, ASDR is projected to continue declining, reaching 48.16 per 100000 population by 2035. A downward trend is also anticipated for ASMR (Supplementary file Figure S7B and S8B). Given that older adults account for the majority of the global dementia burden, age-stratified projections of ASDR among individuals aged ≥40 years were further conducted. The results indicate that smoking-attributable dementia ASDRs are expected to decline across all age groups >40 years (Supplementary file Figure S7C). Among individuals aged 40-75 years, ASDR is projected to decrease markedly, consistent with the trend observed from 1990 to 2021. Projected reductions in ASDR are slower among the 75–79, 80–85, 90–95, and ≥95 years groups, suggesting that the burden in populations aged ≥75 may remain substantial. These age groups warrant focused attention in future dementia prevention efforts.

## DISCUSSION

Among the behavioral, metabolic, and environmental factors implicated in the pathogenesis of AD and other dementias, smoking remains a widely recognized modifiable behavioral risk factor. Consistent with an earlier study^23^ , our findings support that although global ASMR and ASDR of smoking-related AD and other dementias have shown a sustained decline over the past three decades, marked regional disparities in burden remain. Regions with high SDI, which have benefited from earlier and more effective tobacco control measures alongside more comprehensive dementia prevention and care strategies, experienced the most substantial reductions in these rates. Since 2005, the WHO Framework Convention on Tobacco Control has promoted measures such as tobacco taxation and smoke-free public environments^24^ and many high-SDI countries have concurrently advanced dementia prevention efforts, including the integration of cognitive screening and dementia care into national health systems, as exemplified by the United Kingdom^25^. By contrast, several high-middle SDI regions, including Central Asia and Eastern Europe, continue to experience a markedly elevated burden, which appears to be linked to large population size, inadequate implementation of tobacco control initiatives, and lower levels of education^26^, and insufficient healthcare capacity for dementia detection and intervention^2^. Notably, minor fluctuations in ASMR and ASDR observed around 2020 may be associated with disruptions in healthcare delivery during the COVID-19 pandemic. This highlights the importance of health system resilience in sustaining chronic disease management and tobacco control under crisis conditions.

Across the 204 countries and territories analyzed, only 36 showed an increasing trend in ASDR, while most experienced varying degrees of reduction. At the regional level, East Asia had the most pronounced absolute burden in 2021, as evaluated by deaths and DALYs, with China occupying the top position in both indicators. Although ASRs have declined, the country still bears a considerable burden. China, which accounts for the largest share of global tobacco production and consumption, has undergone rapid economic development accompanied by rising purchasing power. Combined with the entrenched role of tobacco in the economic structure, these factors have contributed to delays in the adoption of tobacco control legislation and insufficient enforcement of smoke-free regulations in public settings. Currently, approximately 300 million people in China are smokers, with 96% of them being male^27^. Strengthening tobacco control policies, therefore, remains a priority, alongside improving capacity for dementia screening and intervention. Fiscal measures, such as increasing cigarette prices and tobacco taxes, have been shown to effectively reduce smoking prevalence^28^. Existing evidence further suggests that these policies are associated with lower risks of subjective cognitive decline in older adults^29^, which may ultimately contribute to reductions in dementia incidence at the population level^30^.

In analyses stratified by age and sex, older age groups bore a higher disease burden, reflecting the delayed harmful effects of smoking and increased vulnerability in middle-aged and elderly populations. Consistent with previous studies, heavy smoking in midlife has been shown to elevate the risk of both AD and vascular dementia in males and females across diverse populations^17^. Females generally have a higher risk of developing dementia. The Rotterdam Study reported that the estimated lifetime risk among females was 33%^31^, nearly double that of males. In addition, this difference may be associated with longer life expectancy and declines in estrogen levels during the perimenopausal and postmenopausal periods^32^. In terms of sex distribution, males exhibited consistently higher smoking-attributable dementia deaths and DALYs across all age groups over 40 years, largely due to higher smoking prevalence and earlier initiation. The reduction in ASMR and ASDR was more substantial among females. This reinforces the necessity of integrating sex-based distinctions in behavioral exposure and disease progression into future dementia prevention planning.

Results from the frontier analysis showed that countries with higher SDI, particularly developed nations, generally achieved greater reductions in smoking-attributable dementia burden. In contrast, many high-middle SDI countries have not yet reached the expected control level for their development status. Lebanon ranked first in ASDR among 204 countries and exhibited the largest deviation from the expected frontier value. Previous reports have indicated that transnational tobacco companies have attempted to influence tobacco tax policy in Lebanon^33^, where tobacco tax rates remain well below WHO recommendations and tobacco products are relatively inexpensive. Since 2019, Lebanon has experienced successive national crises, including the COVID-19 pandemic and a severe economic downturn^34,35^. These events, together with a weak public health infrastructure, have constrained the implementation of tobacco control and dementia prevention policies. Lebanon’s performance suggests that in high-middle SDI countries, socio-economic development alone does not necessarily translate into effective policy outcomes, and that enforcement capacity and health resource allocation remain critical determinants.

Health inequality analysis indicated a modest narrowing of socio-economic disparities in smoking-attributable dementia burden over time; however, substantial inequality persists, suggesting that development level continues to shape disease burden. Against this background, decomposition analysis helps explain the divergence between declining rates and increasing absolute burden. Although smoking-attributable ASDR decreased over time, deaths and DALYs continued to rise, driven mainly by population growth and ageing, while epidemiological improvements only partly offset this trend. BAPC projections suggest this divergence will persist through 2035, with deaths and DALYs continuing to increase despite further declines in ASDR and ASMR, highlighting the need to align tobacco control with ageing-responsive prevention and health system planning.

In summary, the findings suggest that efforts to identify and address smoking-related behavioral risk remain insufficient, particularly in low- and middle-SDI countries undergoing demographic transitions, where tobacco control efforts have lagged, and healthcare resources are uneven. Strengthening healthcare coverage, promoting routine screening, and integrating smoking risk control into standard health services may support more effective dementia prevention.

### Limitations

The results should be interpreted within certain limitations. As a secondary analysis of GBD 2021 estimates, the associations are descriptive and cannot establish causality; residual confounding beyond age, sex, and SDI may persist; and smoking attributable burden is derived from modelled risk attribution rather than individual-level data. Data availability, diagnostic capacity, and reporting quality vary across regions, which may affect the completeness and accuracy of burden estimates. In addition, the analysis was limited to smoked tobacco exposure, and projections based on the BAPC model assume continuity of historical trends and do not account for future changes in tobacco control policies or emerging tobacco use patterns.

## CONCLUSIONS

This work examined worldwide dementia burdens attributable to smoking from 1990 to 2021 and projected trends through 2035. Although ASMR and ASDR declined globally, deaths and DALYs continued to increase. The burden remained higher among males and older age groups, with substantial disparities across SDI levels and countries. These findings highlight the potential relevance of integrating tobacco control with strategies that respond to population ageing and strengthen dementia prevention and care capacity to moderate future burden growth.

## Supplementary Material



## Data Availability

The data supporting this research are available from the following source: Global Health Data Exchange (GHDx) query tool (https://vizhub.healthdata.org/gbd-results/).
