# Supplementary material for: Burden of dementia attributable to smoking among adults aged ≥40 years: A secondary dataset analysis of Global Burden of Disease 1990−2021 with projections to 2035
Source: Tob Induc Dis. 2026 May 16;24:10.18332/tid/218789. doi: 10.18332/tid/218789 (PMC13178615; doi:10.18332/tid/218789)
Supplement: Supplementary file 1 [file TID-24-62-s1.pdf]

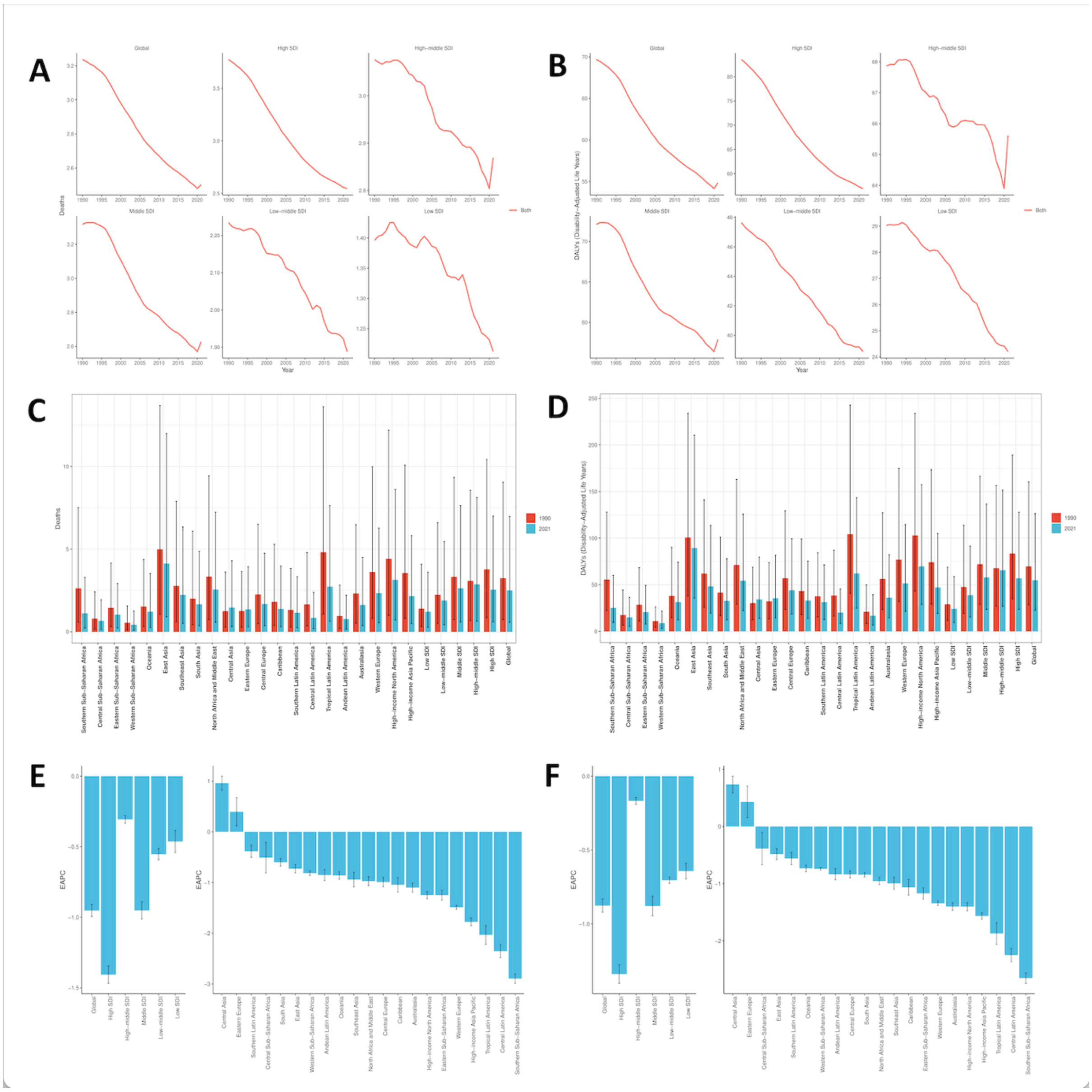

Supplementary file Figure S1. Temporal trends of age-standardised mortality rates (ASMR) (A) and age-standardised disability-adjusted life year rate (ASDR) (B) for dementia attributable to smoking from 1990 to 2021 at the global level and across five sociodemographic index (SDI) regions, based on the Global Burden of Disease (GBD) 2021 study. ASMR (C) and ASDR (D) in 1990 and 2021 at the global level, across five SDI regions and 21 GBD regions. Estimated annual percentage changes (EAPC) in ASMR (E) and ASDR (F) from 1990 to 2021 at the global level and across 21 GBD regions.

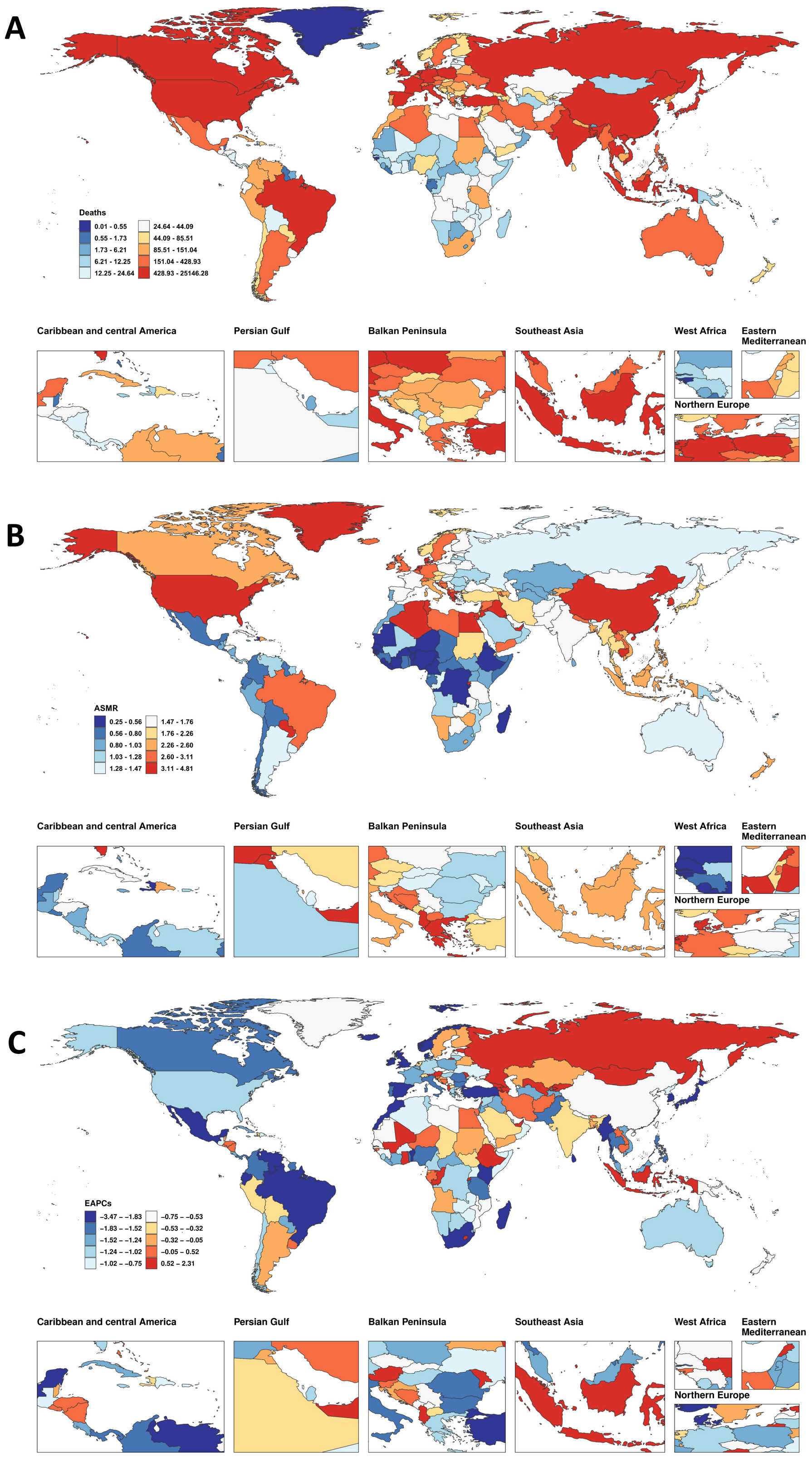

Supplementary file Figure S2. Global map of dementia mortality attributable to smoking in 204 countries and territories, based on the Global Burden of Disease (GBD) 2021 study. (A) Number of deaths in 2021; (B) distribution of age-standardised mortality rates (ASMR) in 2021; (C) estimated annual percentage changes (EAPC) in ASMR from 1990 to 2021.

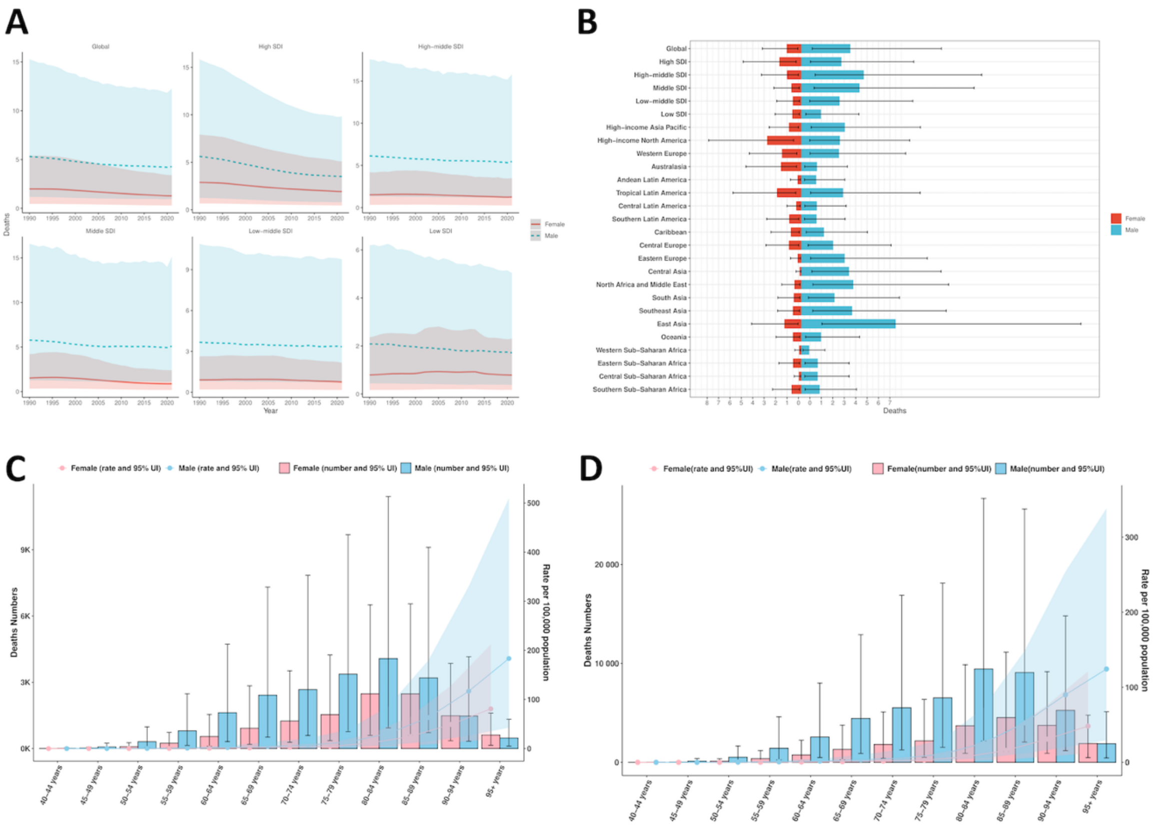

Supplementary file Figure S3. Temporal trends and distributions of deaths from dementia attributable to smoking, by region, age, and sex, based on the Global Burden of Disease (GBD) 2021 study. (A) Temporal trend of age-standardised mortality rates (ASMR) from 1990 to 2021 at the global level and across 21 GBD regions. (B) Distribution of ASMR by sex across 21 GBD regions in 2021. (C) Deaths and ASMR in 1990 by age group and sex. (D) Deaths and ASMR in 2021 by age group and sex. Error bars represent 95% uncertainty intervals (UIs) for deaths; shading represents 95% UIs for rates.

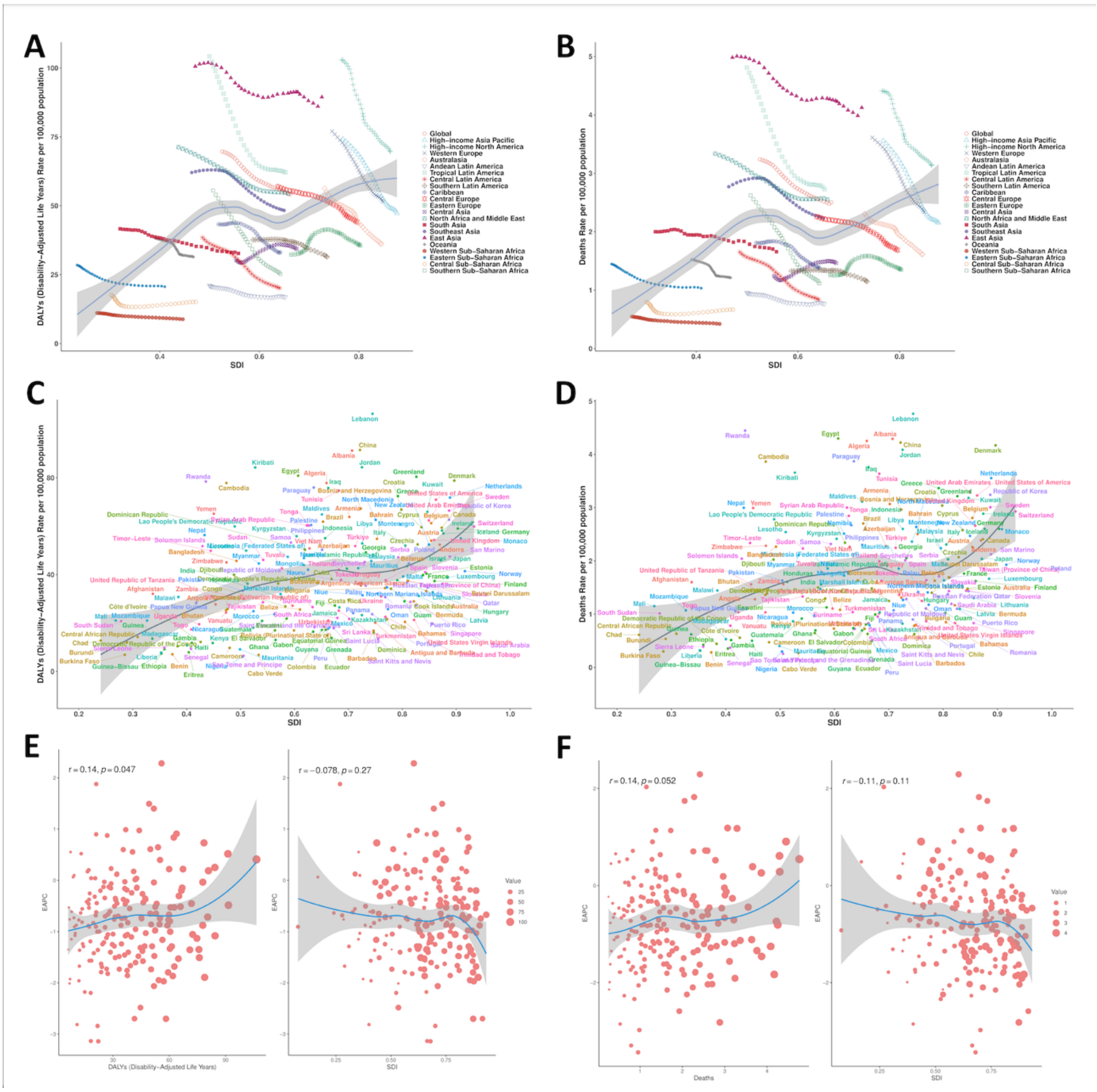

Supplementary file Figure S4. Distribution of age-standardised disability-adjusted life year rates (ASDR) and age-standardised mortality rates (ASMR) for dementia attributable to smoking across sociodemographic index (SDI) levels, based on the Global Burden of Disease (GBD) 2021 study. (A–B) Global and 21 GBD regions from 1990 to 2021; (C–D) 204 countries or territories in 2021. (E–F) Correlation of estimated annual percentage changes (EAPC) with age-standardised rates (ASRs) (left) and SDI (right) in 204 countries and territories in 2021.

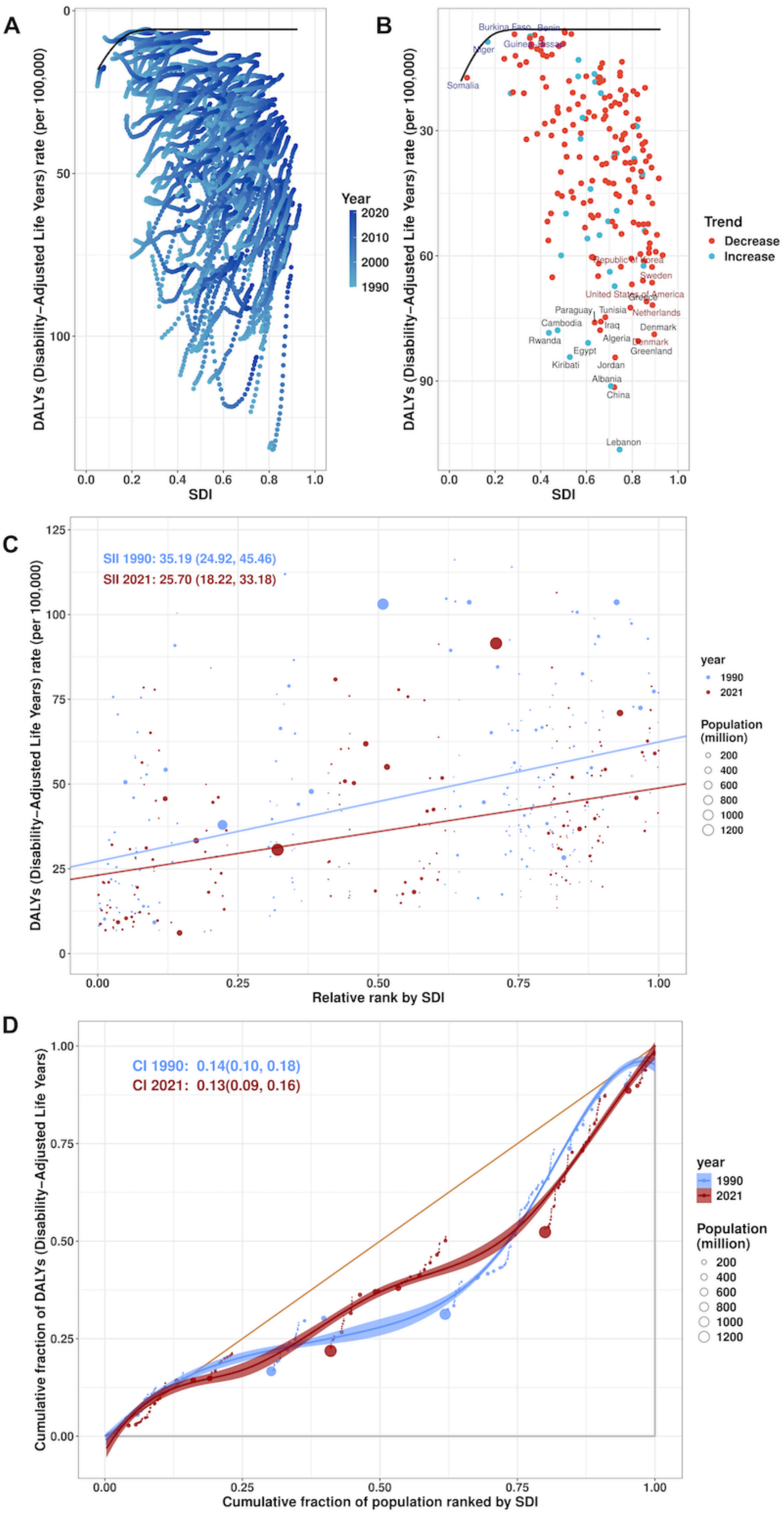

Supplementary file Figure S5. Frontier analysis and health inequality assessment of dementia burden attributable to smoking across sociodemographic index (SDI) levels from 1990 to 2021,

based on the Global Burden of Disease (GBD) 2021 study.

(A) Frontier analysis of age-standardised disability-adjusted life year rate (ASDR) for countries and regions at different development levels from 1990 to 2021, with the efficiency frontier delineated in solid black. Each dot represents a country or region, and the gradient from light to dark blue reflects the combined trajectory of SDI and ASDR over time. (B) Frontier analysis of ASDR for Alzheimer's disease and other dementias attributable to smoking by 2021 SDI values. The top fifteen countries with the largest effective gaps are marked in black. Examples of frontier countries with low SDI (<0.5) and minimal effective gaps are shown in blue, while countries with high SDI (>0.85) but substantial gaps are highlighted in red. (C) Slope index of inequality (SII) showing the association between SDI and ASDR across 204 countries and territories, with population-weighted data points. (D) Concentration curves illustrating socioeconomic inequality by comparing the distribution of ASDR with the equality line, with concentration index values reflecting the degree of deviation.

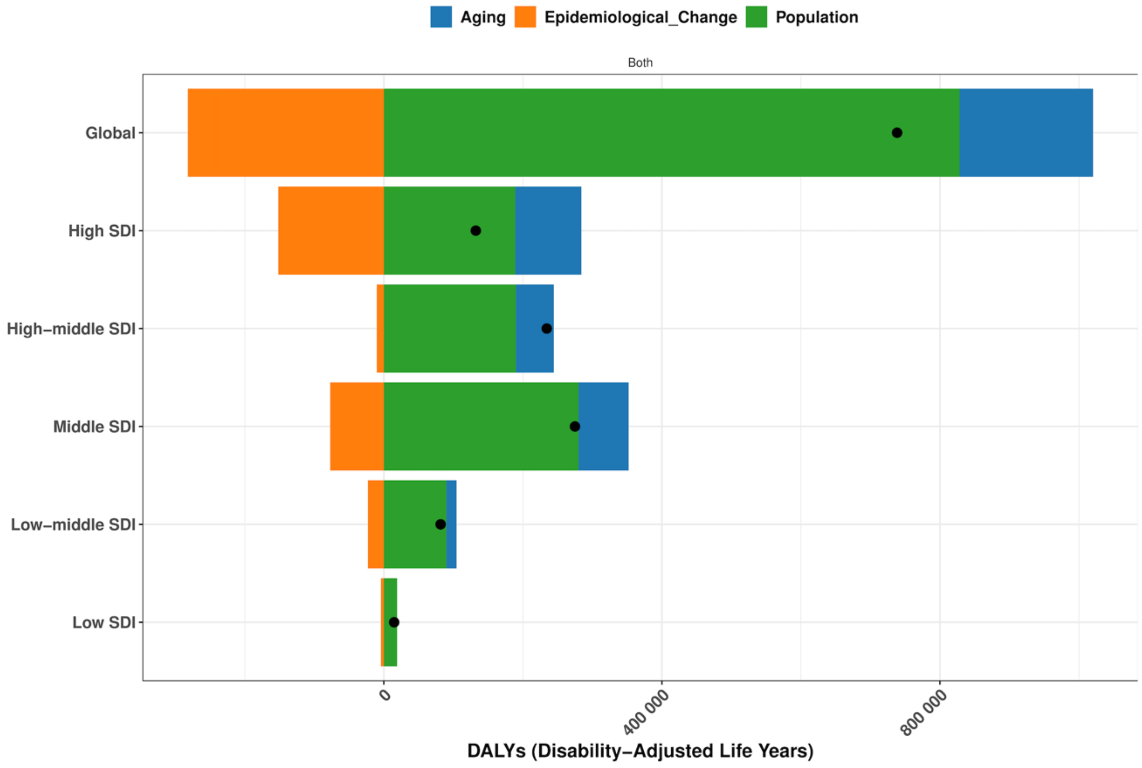

Supplementary file Figure S6. Contributions of population ageing, population growth, and epidemiological changes to the trend in dementia disability-adjusted life years (DALYs) attributable to smoking from 1990 to 2021, based on the Global Burden of Disease (GBD) 2021 study. Black dots represent the total change contributed by all three components. A positive

value for each component indicates an increase in DALYs, whereas a negative value indicates a reduction.

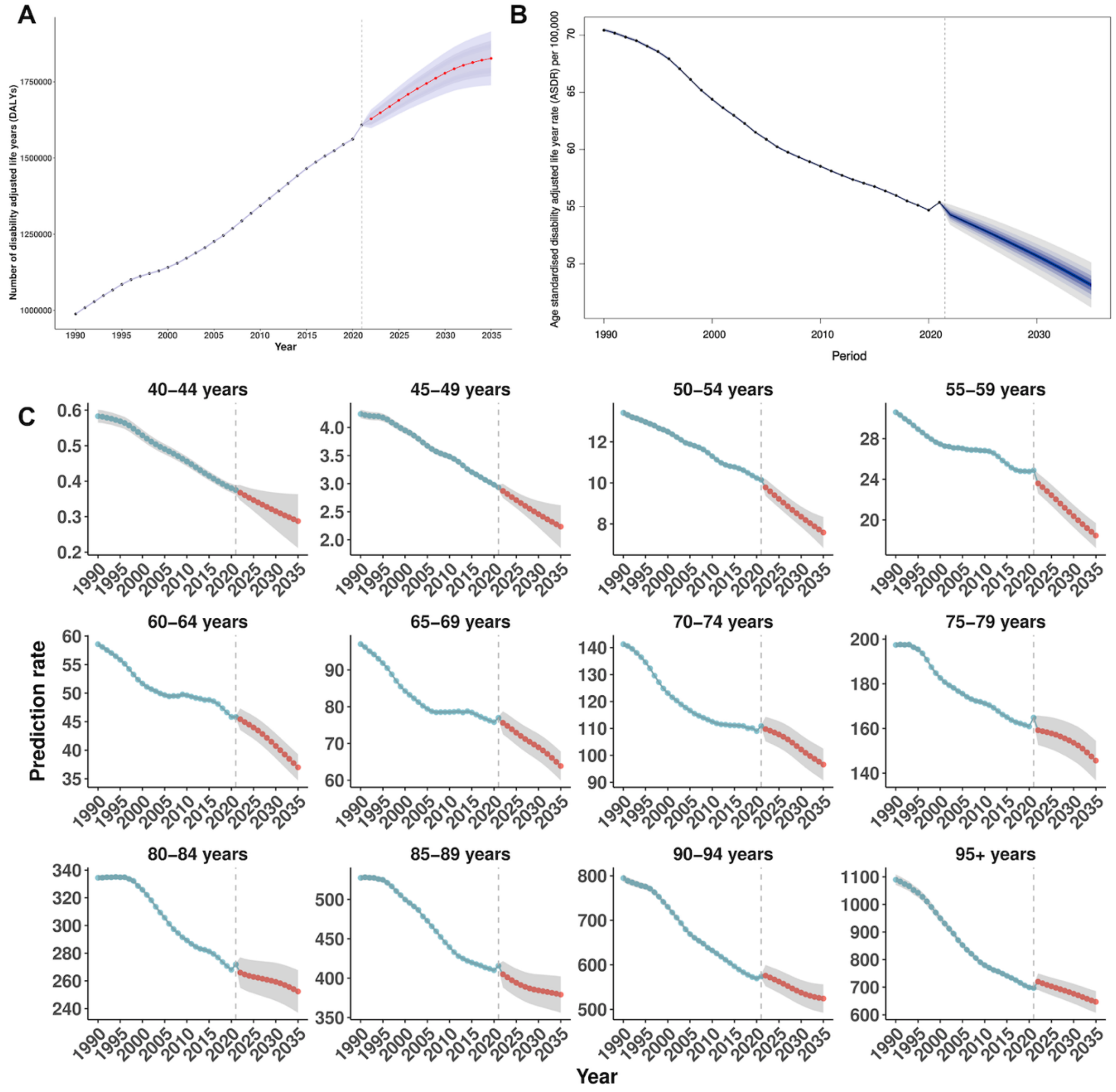

Supplementary file Figure S7. Global trends in dementia burden attributable to smoking from 1990 to 2035 using the Bayesian age–period–cohort (BAPC) model, based on the Global Burden of Disease (GBD) 2021 study: projected disability-adjusted life years (DALYs) (A), age-standardised disability-adjusted life year rate (ASDR) (B), and ASDR by age group among individuals aged 40 years and older (C). The shaded areas represent the 95% confidence intervals (CIs).

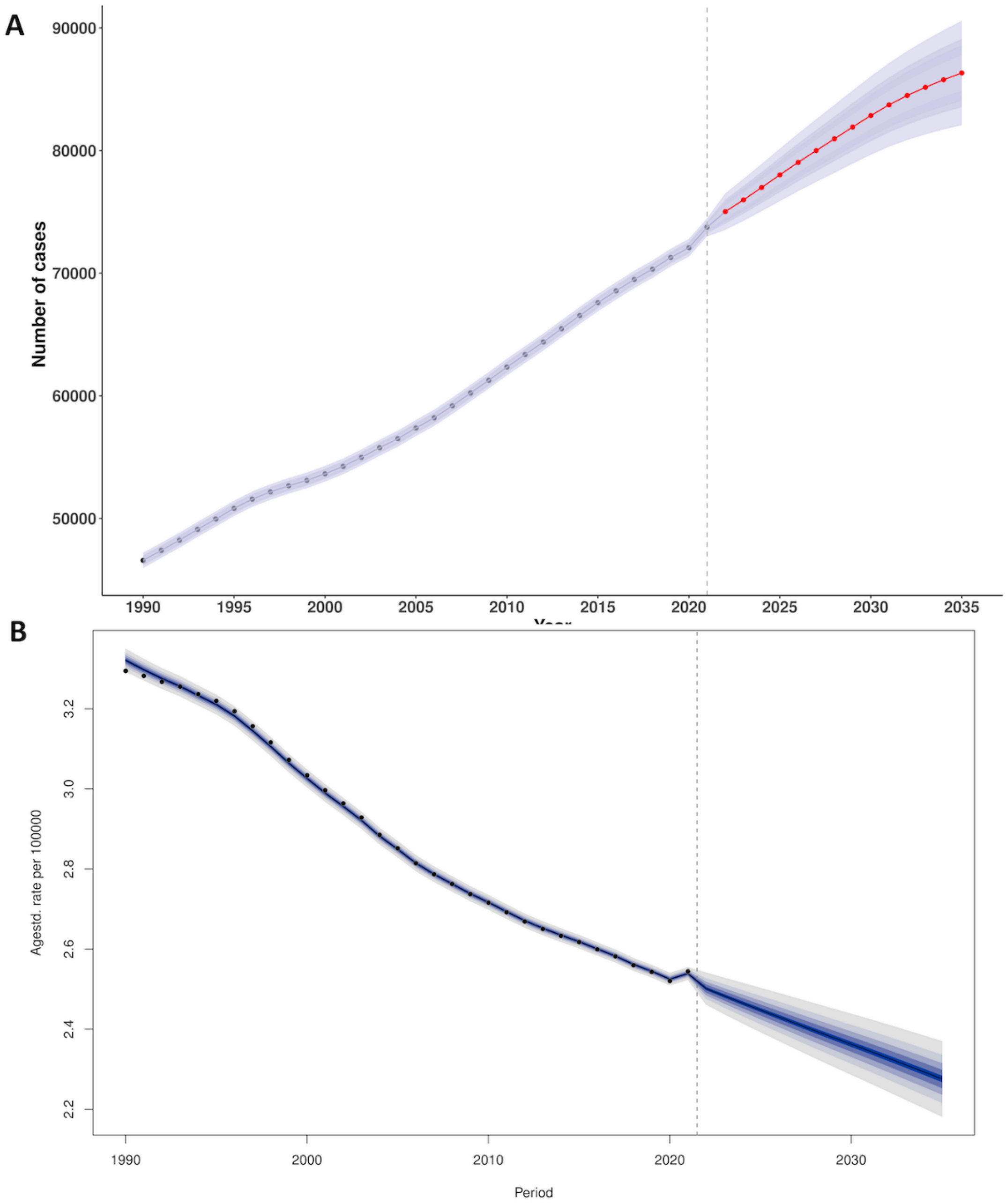

Supplementary file Figure S8. Global trends of deaths (A) and age-standardised mortality rates (ASMR) (B) for dementia attributable to smoking from 1990 to 2035 using the Bayesian age–period–cohort (BAPC) model, based on the Global Burden of Disease (GBD) 2021 study. The shaded areas represent the 95% confidence intervals (CIs).

Table S1. Global deaths and disability-adjusted life years (DALYs) for Alzheimer's disease and other dementias attributable to smoking, 1990–2021, based on the Global Burden of Disease (GBD) 2021 study.

Location	1990		2021	
	**Deaths**	**DALYs**	**Deaths**	**DALYs**
Global	32165(7167, 90875)	794915(328160, 1832306)	67176(15334, 188054)	1533214(635494, 3540712)
**Sex**				
Male	20500(4491, 59321)	532606(218597, 1249854)	46755(10329, 134861)	1110023(455152, 2610152)
Female	11665(2688, 31740)	262310(108907, 587292)	20420(4810, 54048)	423191(174998, 938675)
**Sociodemographic Index**				
Low SDI	560(120, 1649)	15215(6187, 36017)	1216(263, 3650)	30072(11697, 72762)
Low-middle SDI	2734(599, 8145)	71967(29724, 171906)	6466(1418, 18767)	153657(60635, 359920)
Middle SDI	7296(1585, 20848)	195493(79072, 452313)	19215(4233, 56316)	470385(193113, 1110523)
High-middle SDI	7881(1719, 22241)	200798(81328, 463649)	18463(4114, 52435)	435096(178933, 1005900)
High SDI	13666(3109, 37832)	310666(130615, 702662)	21771(5108, 59270)	442915(183731, 995724)
**GBD region**				
Andean Latin America	53(11, 156)	1262(503, 2977)	141(30, 408)	3188(1275, 7443)
Australasia	173(39, 486)	4412(1851, 9949)	337(78, 938)	7016(2816, 16008)
Caribbean	131(27, 385)	3466(1454, 7889)	258(56, 740)	6059(2530, 13803)
Central Asia	161(35, 469)	4360(1806, 9853)	300(64, 887)	8070(3328, 18795)
Central Europe	947(201, 2744)	26911(11210, 60890)	1350(294, 3798)	33947(14360, 76632)
Central Latin America	337(73, 979)	8966(3848, 20171)	655(144, 1865)	16187(6719, 36591)
Central Sub-Saharan Africa	31(6, 97)	964(372, 2414)	72(15, 213)	2170(782, 5238)
East Asia	8275(1752, 23209)	218922(85070, 511412)	25333(5531, 74332)	611761(248099, 1437419)
Eastern Europe	993(208, 2885)	28706(11945, 65340)	1647(351, 4803)	43178(17488, 99434)
Eastern Sub-Saharan Africa	181(39, 528)	4798(1906, 11467)	340(74, 976)	8554(3275, 20523)
High-income Asia Pacific	1982(444, 5668)	45858(18362, 107246)	4738(1101, 12601)	87434(35167, 194159)
High-income North America	5457(1230, 15094)	125527(52921, 285762)	7774(1778, 21207)	164284(67856, 371859)
North Africa and Middle East	1184(256, 3369)	30637(12599, 69931)	2664(586, 7587)	66397(27256, 152854)
Oceania	9(2, 26)	299(116, 712)	20(4, 59)	661(257, 1567)
South Asia	2128(449, 6485)	55864(22839, 134870)	5664(1225, 16651)	129918(49628, 307774)
Southeast Asia	1510(325, 4342)	40981(17306, 93086)	3542(784, 10170)	89722(36338, 211686)
Southern Latin America	185(39, 536)	5659(2339, 12669)	351(75, 1018)	9343(3772, 21209)

Southern Sub-Saharan Africa	168(37, 483)	4087(1664, 9384)	151(31, 445)	4104(1588, 9706)
Tropical Latin America	1028(224, 2951)	26318(10366, 61161)	2220(509, 6217)	52048(20766, 120267)
Western Europe	7135(1620, 19756)	154412(64716, 350463)	9444(2227, 25151)	184594(76702, 411312)
Western Sub-Saharan Africa	98(20, 281)	2506(972, 5941)	175(37, 527)	4580(1722, 11342)

SDI, Sociodemographic index.

Table S2. Number of disability-adjusted life years (DALYs) from smoking-attributable dementia across 204 countries and territories in 2021.

age_name	location_name	cause_name	metric_name	2021-DALYs-Both
All ages	Afghanistan	Alzheimer's disease and other dementias	Number	706,0007503
All ages	Albania	Alzheimer's disease and other dementias	Number	1312,798565
All ages	Algeria	Alzheimer's disease and other dementias	Number	6708,69988
All ages	American Samoa	Alzheimer's disease and other dementias	Number	5,317289374
All ages	Andorra	Alzheimer's disease and other dementias	Number	28,98723777
All ages	Angola	Alzheimer's disease and other dementias	Number	808,5159909
All ages	Antigua and Barbuda	Alzheimer's disease and other dementias	Number	6,744433018
All ages	Argentina	Alzheimer's disease and other dementias	Number	6748,980995
All ages	Armenia	Alzheimer's disease and other dementias	Number	943,733969
All ages	Australia	Alzheimer's disease and other dementias	Number	5419,431981
All ages	Austria	Alzheimer's disease and other dementias	Number	3587,930727
All ages	Azerbaijan	Alzheimer's disease and other dementias	Number	1439,349499
All ages	Bahamas	Alzheimer's disease and other dementias	Number	24,85041377
All ages	Bahrain	Alzheimer's disease and other dementias	Number	109,1859754
All ages	Bangladesh	Alzheimer's disease and other dementias	Number	17971,41322
All ages	Barbados	Alzheimer's disease and other dementias	Number	29,7877895
All ages	Belarus	Alzheimer's disease and other dementias	Number	2322,303474
All ages	Belgium	Alzheimer's disease and other dementias	Number	5504,234643
All ages	Belize	Alzheimer's disease and other dementias	Number	22,54014565
All ages	Benin	Alzheimer's disease and other dementias	Number	106,0909834
All ages	Bermuda	Alzheimer's disease and other dementias	Number	14,30605094
All ages	Bhutan	Alzheimer's disease and other dementias	Number	45,20466445
All ages	Bolivia (Plurinational State of)	Alzheimer's disease and other dementias	Number	456,2671981
All ages	Bosnia and Herzegovina	Alzheimer's disease and other dementias	Number	1436,239974
All ages	Botswana	Alzheimer's disease and other dementias	Number	139,7909929
All ages	Brazil	Alzheimer's disease and other dementias	Number	50696,68515
All ages	Brunei Darussalam	Alzheimer's disease and other dementias	Number	33,26679811
All ages	Bulgaria	Alzheimer's disease and other dementias	Number	1644,161101
All ages	Burkina Faso	Alzheimer's disease and other dementias	Number	179,9725718
All ages	Burundi	Alzheimer's disease and other dementias	Number	153,7619051
All ages	Cabo Verde	Alzheimer's disease and other dementias	Number	10,03429481
All ages	Cambodia	Alzheimer's disease and other dementias	Number	2456,241943
All ages	Cameroon	Alzheimer's disease and other dementias	Number	315,128309
All ages	Canada	Alzheimer's disease and other dementias	Number	15559,38945
All ages	Central African Republic	Alzheimer's disease and other dementias	Number	73,38338808
All ages	Chad	Alzheimer's disease and other dementias	Number	184,8765489
All ages	Chile	Alzheimer's disease and other dementias	Number	1884,456128
All ages	China	Alzheimer's disease and other dementias	Number	602501,0798
All ages	Colombia	Alzheimer's disease and other dementias	Number	3502,710594
All ages	Comoros	Alzheimer's disease and other dementias	Number	32,2153851
All ages	Congo	Alzheimer's disease and other dementias	Number	174,364804
All ages	Cook Islands	Alzheimer's disease and other dementias	Number	2,991592659
All ages	Costa Rica	Alzheimer's disease and other dementias	Number	552,0284933
All ages	Croatia	Alzheimer's disease and other dementias	Number	2162,68015
All ages	Cuba	Alzheimer's disease and other dementias	Number	2621,474041
All ages	Cyprus	Alzheimer's disease and other dementias	Number	419,565455
All ages	Czechia	Alzheimer's disease and other dementias	Number	3954,626616
All ages	Côte d'Ivoire	Alzheimer's disease and other dementias	Number	352,3127354
All ages	Democratic People's Republic of Korea	Alzheimer's disease and other dementias	Number	3774,032408
All ages	Democratic Republic of the Congo	Alzheimer's disease and other dementias	Number	1043,724085
All ages	Denmark	Alzheimer's disease and other dementias	Number	3541,378956
All ages	Djibouti	Alzheimer's disease and other dementias	Number	55,69632539
All ages	Dominica	Alzheimer's disease and other dementias	Number	4,386981521
All ages	Dominican Republic	Alzheimer's disease and other dementias	Number	1601,424979
All ages	Ecuador	Alzheimer's disease and other dementias	Number	782,5627279
All ages	Egypt	Alzheimer's disease and other dementias	Number	10259,33486
All ages	El Salvador	Alzheimer's disease and other dementias	Number	363,4590348
All ages	Equatorial Guinea	Alzheimer's disease and other dementias	Number	22,02839437
All ages	Eritrea	Alzheimer's disease and other dementias	Number	56,82288698
All ages	Estonia	Alzheimer's disease and other dementias	Number	384,7772829
All ages	Eswatini	Alzheimer's disease and other dementias	Number	25,90232737
All ages	Ethiopia	Alzheimer's disease and other dementias	Number	1108,924713
All ages	Fiji	Alzheimer's disease and other dementias	Number	64,27662047
All ages	Finland	Alzheimer's disease and other dementias	Number	1750,314421
All ages	France	Alzheimer's disease and other dementias	Number	21153,2994
All ages	Gabon	Alzheimer's disease and other dementias	Number	48,12799029
All ages	Gambia	Alzheimer's disease and other dementias	Number	32,84175204
All ages	Georgia	Alzheimer's disease and other dementias	Number	1062,025557
All ages	Germany	Alzheimer's disease and other dementias	Number	44416,01125
All ages	Ghana	Alzheimer's disease and other dementias	Number	500,198781
All ages	Greece	Alzheimer's disease and other dementias	Number	7055,371409
All ages	Greenland	Alzheimer's disease and other dementias	Number	16,55191267
All ages	Grenada	Alzheimer's disease and other dementias	Number	4,978522299
All ages	Guam	Alzheimer's disease and other dementias	Number	21,59890961
All ages	Guatemala	Alzheimer's disease and other dementias	Number	691,0574499
All ages	Guinea	Alzheimer's disease and other dementias	Number	212,4381823
All ages	Guinea-Bissau	Alzheimer's disease and other dementias	Number	14,11249552
All ages	Guyana	Alzheimer's disease and other dementias	Number	31,9482726
All ages	Haiti	Alzheimer's disease and other dementias	Number	216,6710344
All ages	Honduras	Alzheimer's disease and other dementias	Number	656,490472
All ages	Hungary	Alzheimer's disease and other dementias	Number	2527,396474
All ages	Iceland	Alzheimer's disease and other dementias	Number	122,7822098
All ages	India	Alzheimer's disease and other dementias	Number	98343,85986
All ages	Indonesia	Alzheimer's disease and other dementias	Number	32681,8956
All ages	Iran (Islamic Republic of)	Alzheimer's disease and other dementias	Number	9751,682428
All ages	Iraq	Alzheimer's disease and other dementias	Number	4309,339782
All ages	Ireland	Alzheimer's disease and other dementias	Number	1646,871782
All ages	Israel	Alzheimer's disease and other dementias	Number	1939,08867
All ages	Italy	Alzheimer's disease and other dementias	Number	29262,67349
All ages	Jamaica	Alzheimer's disease and other dementias	Number	287,7161259
All ages	Japan	Alzheimer's disease and other dementias	Number	67192,09023
All ages	Jordan	Alzheimer's disease and other dementias	Number	1491,519505
All ages	Kazakhstan	Alzheimer's disease and other dementias	Number	1311,655709
All ages	Kenya	Alzheimer's disease and other dementias	Number	1056,806723
All ages	Kiribati	Alzheimer's disease and other dementias	Number	15,50526948
All ages	Kuwait	Alzheimer's disease and other dementias	Number	475,3182452
All ages	Kyrgyzstan	Alzheimer's disease and other dementias	Number	752,952512
All ages	Lao People's Democratic Republic	Alzheimer's disease and other dementias	Number	695,4300877
All ages	Latvia	Alzheimer's disease and other dementias	Number	432,6195013
All ages	Lebanon	Alzheimer's disease and other dementias	Number	2291,512213
All ages	Lesotho	Alzheimer's disease and other dementias	Number	120,547233
All ages	Liberia	Alzheimer's disease and other dementias	Number	46,90464646
All ages	Libya	Alzheimer's disease and other dementias	Number	740,2711791
All ages	Lithuania	Alzheimer's disease and other dementias	Number	690,6993004
All ages	Luxembourg	Alzheimer's disease and other dementias	Number	146,3636935
All ages	Madagascar	Alzheimer's disease and other dementias	Number	254,8848752
All ages	Malawi	Alzheimer's disease and other dementias	Number	573,246042
All ages	Malaysia	Alzheimer's disease and other dementias	Number	3533,26626
All ages	Maldives	Alzheimer's disease and other dementias	Number	58,24225615
All ages	Mali	Alzheimer's disease and other dementias	Number	422,362152
All ages	Malta	Alzheimer's disease and other dementias	Number	137,4474008
All ages	Marshall Islands	Alzheimer's disease and other dementias	Number	2,657162843
All ages	Mauritania	Alzheimer's disease and other dementias	Number	58,45281785
All ages	Mauritius	Alzheimer's disease and other dementias	Number	253,7082207
All ages	Mexico	Alzheimer's disease and other dementias	Number	7064,798511
All ages	Micronesia (Federated States of)	Alzheimer's disease and other dementias	Number	10,43632482
All ages	Monaco	Alzheimer's disease and other dementias	Number	20,8235946
All ages	Mongolia	Alzheimer's disease and other dementias	Number	272,6892343
All ages	Montenegro	Alzheimer's disease and other dementias	Number	199,2062659
All ages	Morocco	Alzheimer's disease and other dementias	Number	2499,985879
All ages	Mozambique	Alzheimer's disease and other dementias	Number	551,3461264
All ages	Myanmar	Alzheimer's disease and other dementias	Number	5923,784755
All ages	Namibia	Alzheimer's disease and other dementias	Number	146,7232773
All ages	Nauru	Alzheimer's disease and other dementias	Number	0,643673973
All ages	Nepal	Alzheimer's disease and other dementias	Number	3349,844945
All ages	Netherlands	Alzheimer's disease and other dementias	Number	9399,82832
All ages	New Zealand	Alzheimer's disease and other dementias	Number	1596,236525
All ages	Nicaragua	Alzheimer's disease and other dementias	Number	366,9352014
All ages	Niger	Alzheimer's disease and other dementias	Number	145,5642147
All ages	Nigeria	Alzheimer's disease and other dementias	Number	1489,115325
All ages	Niue	Alzheimer's disease and other dementias	Number	0,228472873
All ages	North Macedonia	Alzheimer's disease and other dementias	Number	607,3957699
All ages	Northern Mariana Islands	Alzheimer's disease and other dementias	Number	5,38039778
All ages	Norway	Alzheimer's disease and other dementias	Number	1538,490041
All ages	Oman	Alzheimer's disease and other dementias	Number	122,7379196
All ages	Pakistan	Alzheimer's disease and other dementias	Number	10207,76577
All ages	Palau	Alzheimer's disease and other dementias	Number	1,990111012
All ages	Palestine	Alzheimer's disease and other dementias	Number	395,1475355
All ages	Panama	Alzheimer's disease and other dementias	Number	365,1149643
All ages	Papua New Guinea	Alzheimer's disease and other dementias	Number	401,0492163
All ages	Paraguay	Alzheimer's disease and other dementias	Number	1351,567248
All ages	Peru	Alzheimer's disease and other dementias	Number	1948,759638
All ages	Philippines	Alzheimer's disease and other dementias	Number	11239,33803
All ages	Poland	Alzheimer's disease and other dementias	Number	11161,02805
All ages	Portugal	Alzheimer's disease and other dementias	Number	2052,252098
All ages	Puerto Rico	Alzheimer's disease and other dementias	Number	748,2867779
All ages	Qatar	Alzheimer's disease and other dementias	Number	78,41975539
All ages	Republic of Korea	Alzheimer's disease and other dementias	Number	19643,64115
All ages	Republic of Moldova	Alzheimer's disease and other dementias	Number	728,5816969
All ages	Romania	Alzheimer's disease and other dementias	Number	3927,331516
All ages	Russian Federation	Alzheimer's disease and other dementias	Number	30294,43101
All ages	Rwanda	Alzheimer's disease and other dementias	Number	1071,426336
All ages	Saint Kitts and Nevis	Alzheimer's disease and other dementias	Number	2,651503701
All ages	Saint Lucia	Alzheimer's disease and other dementias	Number	14,2529746
All ages	Saint Vincent and the Grenadines	Alzheimer's disease and other dementias	Number	8,528691182
All ages	Samoa	Alzheimer's disease and other dementias	Number	22,77841874
All ages	San Marino	Alzheimer's disease and other dementias	Number	14,4021458
All ages	Sao Tome and Principe	Alzheimer's disease and other dementias	Number	2,181826154
All ages	Saudi Arabia	Alzheimer's disease and other dementias	Number	1350,145533
All ages	Senegal	Alzheimer's disease and other dementias	Number	224,7895682
All ages	Serbia	Alzheimer's disease and other dementias	Number	2677,080719
All ages	Seychelles	Alzheimer's disease and other dementias	Number	15,63891281
All ages	Sierra Leone	Alzheimer's disease and other dementias	Number	108,8271848
All ages	Singapore	Alzheimer's disease and other dementias	Number	564,8388432
All ages	Slovakia	Alzheimer's disease and other dementias	Number	1211,31195
All ages	Slovenia	Alzheimer's disease and other dementias	Number	631,8264175
All ages	Solomon Islands	Alzheimer's disease and other dementias	Number	48,55912185
All ages	Somalia	Alzheimer's disease and other dementias	Number	233,4054379
All ages	South Africa	Alzheimer's disease and other dementias	Number	2993,22981
All ages	South Sudan	Alzheimer's disease and other dementias	Number	192,6274144
All ages	Spain	Alzheimer's disease and other dementias	Number	13722,94
All ages	Sri Lanka	Alzheimer's disease and other dementias	Number	1775,166043
All ages	Sudan	Alzheimer's disease and other dementias	Number	2257,807957
All ages	Suriname	Alzheimer's disease and other dementias	Number	54,23758446
All ages	Sweden	Alzheimer's disease and other dementias	Number	5610,531052
All ages	Switzerland	Alzheimer's disease and other dementias	Number	4289,452971
All ages	Syrian Arab Republic	Alzheimer's disease and other dementias	Number	1959,549343
All ages	Taiwan (Province of China)	Alzheimer's disease and other dementias	Number	5485,409593
All ages	Tajikistan	Alzheimer's disease and other dementias	Number	426,1492937
All ages	Thailand	Alzheimer's disease and other dementias	Number	15621,96499
All ages	Timor-Leste	Alzheimer's disease and other dementias	Number	112,3318979
All ages	Togo	Alzheimer's disease and other dementias	Number	173,5718955
All ages	Tokelau	Alzheimer's disease and other dementias	Number	0,188861657
All ages	Tonga	Alzheimer's disease and other dementias	Number	15,25473039
All ages	Trinidad and Tobago	Alzheimer's disease and other dementias	Number	145,9128115
All ages	Tunisia	Alzheimer's disease and other dementias	Number	2973,187814
All ages	Turkey	Alzheimer's disease and other dementias	Number	15189,13441
All ages	Turkmenistan	Alzheimer's disease and other dementias	Number	290,0600986
All ages	Tuvalu	Alzheimer's disease and other dementias	Number	1,34111542
All ages	Uganda	Alzheimer's disease and other dementias	Number	681,7695059
All ages	Ukraine	Alzheimer's disease and other dementias	Number	8324,271007
All ages	United Arab Emirates	Alzheimer's disease and other dementias	Number	373,86534
All ages	United Kingdom	Alzheimer's disease and other dementias	Number	27070,10646
All ages	United Republic of Tanzania	Alzheimer's disease and other dementias	Number	2071,750627
All ages	United States of America	Alzheimer's disease and other dementias	Number	148705,2223
All ages	United States Virgin Islands	Alzheimer's disease and other dementias	Number	13,73655672
All ages	Uruguay	Alzheimer's disease and other dementias	Number	709,1754837
All ages	Uzbekistan	Alzheimer's disease and other dementias	Number	1571,165887
All ages	Vanuatu	Alzheimer's disease and other dementias	Number	11,21510439
All ages	Venezuela (Bolivarian Republic of)	Alzheimer's disease and other dementias	Number	2624,221357
All ages	Viet Nam	Alzheimer's disease and other dementias	Number	15230,13425
All ages	Yemen	Alzheimer's disease and other dementias	Number	2292,01843
All ages	Zambia	Alzheimer's disease and other dementias	Number	451,871208
All ages	Zimbabwe	Alzheimer's disease and other dementias	Number	677,719199

Table S2. Age-standardised mortality rates (ASMR) from smoking-attributable dementia across 204 countries and territories in 2021.

age_name	location_name	cause_name	metric_name	2021-ASMR-Both
All ages	Afghanistan	Alzheimer's disease and other dementias	Rate	1,600710229
All ages	Albania	Alzheimer's disease and other dementias	Rate	4,292048437
All ages	Algeria	Alzheimer's disease and other dementias	Rate	4,251807611
All ages	American Samoa	Alzheimer's disease and other dementias	Rate	1,465715341
All ages	Andorra	Alzheimer's disease and other dementias	Rate	2,383888045
All ages	Angola	Alzheimer's disease and other dementias	Rate	1,281675232
All ages	Antigua and Barbuda	Alzheimer's disease and other dementias	Rate	0,800745363
All ages	Argentina	Alzheimer's disease and other dementias	Rate	1,308828921
All ages	Armenia	Alzheimer's disease and other dementias	Rate	3,068044977
All ages	Australia	Alzheimer's disease and other dementias	Rate	1,463198413
All ages	Austria	Alzheimer's disease and other dementias	Rate	2,258543772
All ages	Azerbaijan	Alzheimer's disease and other dementias	Rate	2,278328024
All ages	Bahamas	Alzheimer's disease and other dementias	Rate	0,850797094
All ages	Bahrain	Alzheimer's disease and other dementias	Rate	2,50456963
All ages	Bangladesh	Alzheimer's disease and other dementias	Rate	2,267667744
All ages	Barbados	Alzheimer's disease and other dementias	Rate	0,782486468
All ages	Belarus	Alzheimer's disease and other dementias	Rate	1,627967476
All ages	Belgium	Alzheimer's disease and other dementias	Rate	2,806706523
All ages	Belize	Alzheimer's disease and other dementias	Rate	1,082443937
All ages	Benin	Alzheimer's disease and other dementias	Rate	0,283003928
All ages	Bermuda	Alzheimer's disease and other dementias	Rate	1,168878489
All ages	Bhutan	Alzheimer's disease and other dementias	Rate	1,362506501
All ages	Bolivia (Plurinational State of)	Alzheimer's disease and other dementias	Rate	0,729972708
All ages	Bosnia and Herzegovina	Alzheimer's disease and other dementias	Rate	2,963679481
All ages	Botswana	Alzheimer's disease and other dementias	Rate	1,632620601
All ages	Brazil	Alzheimer's disease and other dementias	Rate	2,703114025
All ages	Brunei Darussalam	Alzheimer's disease and other dementias	Rate	1,690261144
All ages	Bulgaria	Alzheimer's disease and other dementias	Rate	1,105466321
All ages	Burkina Faso	Alzheimer's disease and other dementias	Rate	0,300269231
All ages	Burundi	Alzheimer's disease and other dementias	Rate	0,537332
All ages	Cabo Verde	Alzheimer's disease and other dementias	Rate	0,270192729
All ages	Cambodia	Alzheimer's disease and other dementias	Rate	3,864036514
All ages	Cameroon	Alzheimer's disease and other dementias	Rate	0,473097554
All ages	Canada	Alzheimer's disease and other dementias	Rate	2,408437412
All ages	Central African Republic	Alzheimer's disease and other dementias	Rate	0,633470589
All ages	Chad	Alzheimer's disease and other dementias	Rate	0,612929224
All ages	Chile	Alzheimer's disease and other dementias	Rate	0,73763939
All ages	China	Alzheimer's disease and other dementias	Rate	4,222636713
All ages	Colombia	Alzheimer's disease and other dementias	Rate	0,725873608
All ages	Comoros	Alzheimer's disease and other dementias	Rate	1,390416313
All ages	Congo	Alzheimer's disease and other dementias	Rate	1,2953668
All ages	Cook Islands	Alzheimer's disease and other dementias	Rate	1,401907249
All ages	Costa Rica	Alzheimer's disease and other dementias	Rate	1,248568443
All ages	Croatia	Alzheimer's disease and other dementias	Rate	3,038166579
All ages	Cuba	Alzheimer's disease and other dementias	Rate	1,483029769
All ages	Cyprus	Alzheimer's disease and other dementias	Rate	2,57826263
All ages	Czechia	Alzheimer's disease and other dementias	Rate	2,071012226
All ages	Côte d'Ivoire	Alzheimer's disease and other dementias	Rate	0,557711804
All ages	Democratic People's Republic of Korea	Alzheimer's disease and other dementias	Rate	1,499158048
All ages	Democratic Republic of the Congo	Alzheimer's disease and other dementias	Rate	0,44358419
All ages	Denmark	Alzheimer's disease and other dementias	Rate	4,16913662
All ages	Djibouti	Alzheimer's disease and other dementias	Rate	2,025136138
All ages	Dominica	Alzheimer's disease and other dementias	Rate	0,625550931
All ages	Dominican Republic	Alzheimer's disease and other dementias	Rate	2,34246716
All ages	Ecuador	Alzheimer's disease and other dementias	Rate	0,630001292
All ages	Egypt	Alzheimer's disease and other dementias	Rate	4,298202965
All ages	El Salvador	Alzheimer's disease and other dementias	Rate	0,645368107
All ages	Equatorial Guinea	Alzheimer's disease and other dementias	Rate	0,806657896
All ages	Eritrea	Alzheimer's disease and other dementias	Rate	0,354207885
All ages	Estonia	Alzheimer's disease and other dementias	Rate	1,477982712
All ages	Eswatini	Alzheimer's disease and other dementias	Rate	1,12771789
All ages	Ethiopia	Alzheimer's disease and other dementias	Rate	0,425022613
All ages	Fiji	Alzheimer's disease and other dementias	Rate	0,993795816
All ages	Finland	Alzheimer's disease and other dementias	Rate	1,621843213
All ages	France	Alzheimer's disease and other dementias	Rate	1,761279682
All ages	Gabon	Alzheimer's disease and other dementias	Rate	0,737802562
All ages	Gambia	Alzheimer's disease and other dementias	Rate	0,514651745
All ages	Georgia	Alzheimer's disease and other dementias	Rate	2,053758796
All ages	Germany	Alzheimer's disease and other dementias	Rate	2,601804231
All ages	Ghana	Alzheimer's disease and other dementias	Rate	0,696664123
All ages	Greece	Alzheimer's disease and other dementias	Rate	3,366371876
All ages	Greenland	Alzheimer's disease and other dementias	Rate	3,21462076
All ages	Grenada	Alzheimer's disease and other dementias	Rate	0,50294108
All ages	Guam	Alzheimer's disease and other dementias	Rate	1,023416466
All ages	Guatemala	Alzheimer's disease and other dementias	Rate	0,919220734
All ages	Guinea	Alzheimer's disease and other dementias	Rate	0,621835826
All ages	Guinea-Bissau	Alzheimer's disease and other dementias	Rate	0,3153605
All ages	Guyana	Alzheimer's disease and other dementias	Rate	0,697057302
All ages	Haiti	Alzheimer's disease and other dementias	Rate	0,502622247
All ages	Honduras	Alzheimer's disease and other dementias	Rate	1,606527881
All ages	Hungary	Alzheimer's disease and other dementias	Rate	1,3308708
All ages	Iceland	Alzheimer's disease and other dementias	Rate	2,602085422
All ages	India	Alzheimer's disease and other dementias	Rate	1,55857775
All ages	Indonesia	Alzheimer's disease and other dementias	Rate	2,592047406
All ages	Iran (Islamic Republic of)	Alzheimer's disease and other dementias	Rate	1,913372999
All ages	Iraq	Alzheimer's disease and other dementias	Rate	3,75996946
All ages	Ireland	Alzheimer's disease and other dementias	Rate	2,885370919
All ages	Israel	Alzheimer's disease and other dementias	Rate	2,03058599
All ages	Italy	Alzheimer's disease and other dementias	Rate	2,308156633
All ages	Jamaica	Alzheimer's disease and other dementias	Rate	1,033558324
All ages	Japan	Alzheimer's disease and other dementias	Rate	2,038128552
All ages	Jordan	Alzheimer's disease and other dementias	Rate	4,085839727
All ages	Kazakhstan	Alzheimer's disease and other dementias	Rate	0,821762709
All ages	Kenya	Alzheimer's disease and other dementias	Rate	0,866613597
All ages	Kiribati	Alzheimer's disease and other dementias	Rate	3,657390234
All ages	Kuwait	Alzheimer's disease and other dementias	Rate	3,182519041
All ages	Kyrgyzstan	Alzheimer's disease and other dementias	Rate	2,413658284
All ages	Lao People's Democratic Republic	Alzheimer's disease and other dementias	Rate	2,781065495
All ages	Latvia	Alzheimer's disease and other dementias	Rate	1,099054009
All ages	Lebanon	Alzheimer's disease and other dementias	Rate	4,76239577
All ages	Lesotho	Alzheimer's disease and other dementias	Rate	2,5415326
All ages	Liberia	Alzheimer's disease and other dementias	Rate	0,321412879
All ages	Libya	Alzheimer's disease and other dementias	Rate	2,600747335
All ages	Lithuania	Alzheimer's disease and other dementias	Rate	1,303100404
All ages	Luxembourg	Alzheimer's disease and other dementias	Rate	1,665755973
All ages	Madagascar	Alzheimer's disease and other dementias	Rate	0,531896129
All ages	Malawi	Alzheimer's disease and other dementias	Rate	1,444643402
All ages	Malaysia	Alzheimer's disease and other dementias	Rate	2,361808342
All ages	Maldives	Alzheimer's disease and other dementias	Rate	3,112765504
All ages	Mali	Alzheimer's disease and other dementias	Rate	1,152074258
All ages	Malta	Alzheimer's disease and other dementias	Rate	1,634296087
All ages	Marshall Islands	Alzheimer's disease and other dementias	Rate	1,472386901
All ages	Mauritania	Alzheimer's disease and other dementias	Rate	0,401030184
All ages	Mauritius	Alzheimer's disease and other dementias	Rate	1,856343946
All ages	Mexico	Alzheimer's disease and other dementias	Rate	0,79156067
All ages	Micronesia (Federated States of)	Alzheimer's disease and other dementias	Rate	1,857013993
All ages	Monaco	Alzheimer's disease and other dementias	Rate	2,596510322
All ages	Mongolia	Alzheimer's disease and other dementias	Rate	1,725815432
All ages	Montenegro	Alzheimer's disease and other dementias	Rate	2,238634103
All ages	Morocco	Alzheimer's disease and other dementias	Rate	0,966110061
All ages	Mozambique	Alzheimer's disease and other dementias	Rate	1,073101109
All ages	Myanmar	Alzheimer's disease and other dementias	Rate	2,106615872
All ages	Namibia	Alzheimer's disease and other dementias	Rate	2,511784029
All ages	Nauru	Alzheimer's disease and other dementias	Rate	1,76321831
All ages	Nepal	Alzheimer's disease and other dementias	Rate	2,997237917
All ages	Netherlands	Alzheimer's disease and other dementias	Rate	3,554672912
All ages	New Zealand	Alzheimer's disease and other dementias	Rate	2,468294887
All ages	Nicaragua	Alzheimer's disease and other dementias	Rate	1,029416377
All ages	Niger	Alzheimer's disease and other dementias	Rate	0,50985429
All ages	Nigeria	Alzheimer's disease and other dementias	Rate	0,298803483
All ages	Niue	Alzheimer's disease and other dementias	Rate	1,267948455
All ages	North Macedonia	Alzheimer's disease and other dementias	Rate	2,637349113
All ages	Northern Mariana Islands	Alzheimer's disease and other dementias	Rate	1,407995376
All ages	Norway	Alzheimer's disease and other dementias	Rate	1,92790677
All ages	Oman	Alzheimer's disease and other dementias	Rate	1,177039838
All ages	Pakistan	Alzheimer's disease and other dementias	Rate	1,631107486
All ages	Palau	Alzheimer's disease and other dementias	Rate	1,415201476
All ages	Palestine	Alzheimer's disease and other dementias	Rate	2,669935212
All ages	Panama	Alzheimer's disease and other dementias	Rate	1,073090402
All ages	Papua New Guinea	Alzheimer's disease and other dementias	Rate	1,056582851
All ages	Paraguay	Alzheimer's disease and other dementias	Rate	3,870933308
All ages	Peru	Alzheimer's disease and other dementias	Rate	0,824218858
All ages	Philippines	Alzheimer's disease and other dementias	Rate	2,302299414
All ages	Poland	Alzheimer's disease and other dementias	Rate	1,690828093
All ages	Portugal	Alzheimer's disease and other dementias	Rate	0,901054642
All ages	Puerto Rico	Alzheimer's disease and other dementias	Rate	1,101899825
All ages	Qatar	Alzheimer's disease and other dementias	Rate	1,432005907
All ages	Republic of Korea	Alzheimer's disease and other dementias	Rate	3,241705342
All ages	Republic of Moldova	Alzheimer's disease and other dementias	Rate	1,284220406
All ages	Romania	Alzheimer's disease and other dementias	Rate	1,108044365
All ages	Russian Federation	Alzheimer's disease and other dementias	Rate	1,373781648
All ages	Rwanda	Alzheimer's disease and other dementias	Rate	4,447451804
All ages	Saint Kitts and Nevis	Alzheimer's disease and other dementias	Rate	0,559550478
All ages	Saint Lucia	Alzheimer's disease and other dementias	Rate	0,717485563
All ages	Saint Vincent and the Grenadines	Alzheimer's disease and other dementias	Rate	0,723685865
All ages	Samoa	Alzheimer's disease and other dementias	Rate	2,31860798
All ages	San Marino	Alzheimer's disease and other dementias	Rate	2,124963637
All ages	Sao Tome and Principe	Alzheimer's disease and other dementias	Rate	0,256220187
All ages	Saudi Arabia	Alzheimer's disease and other dementias	Rate	1,193212084
All ages	Senegal	Alzheimer's disease and other dementias	Rate	0,371948141
All ages	Serbia	Alzheimer's disease and other dementias	Rate	1,70937141
All ages	Seychelles	Alzheimer's disease and other dementias	Rate	2,011804885
All ages	Sierra Leone	Alzheimer's disease and other dementias	Rate	0,389289581
All ages	Singapore	Alzheimer's disease and other dementias	Rate	0,832452536
All ages	Slovakia	Alzheimer's disease and other dementias	Rate	1,432743568
All ages	Slovenia	Alzheimer's disease and other dementias	Rate	1,439978084
All ages	Solomon Islands	Alzheimer's disease and other dementias	Rate	2,04221138
All ages	Somalia	Alzheimer's disease and other dementias	Rate	0,798522293
All ages	South Africa	Alzheimer's disease and other dementias	Rate	0,954255388
All ages	South Sudan	Alzheimer's disease and other dementias	Rate	1,0149199
All ages	Spain	Alzheimer's disease and other dementias	Rate	1,639648079
All ages	Sri Lanka	Alzheimer's disease and other dementias	Rate	0,985504174
All ages	Sudan	Alzheimer's disease and other dementias	Rate	2,233250014
All ages	Suriname	Alzheimer's disease and other dementias	Rate	1,037898046
All ages	Sweden	Alzheimer's disease and other dementias	Rate	3,006684843
All ages	Switzerland	Alzheimer's disease and other dementias	Rate	2,928730882
All ages	Syrian Arab Republic	Alzheimer's disease and other dementias	Rate	3,000650624
All ages	Taiwan (Province of China)	Alzheimer's disease and other dementias	Rate	1,858096921
All ages	Tajikistan	Alzheimer's disease and other dementias	Rate	1,332302905
All ages	Thailand	Alzheimer's disease and other dementias	Rate	1,994415079
All ages	Timor-Leste	Alzheimer's disease and other dementias	Rate	2,336824617
All ages	Togo	Alzheimer's disease and other dementias	Rate	0,782828969
All ages	Tokelau	Alzheimer's disease and other dementias	Rate	1,62590438
All ages	Tonga	Alzheimer's disease and other dementias	Rate	2,846014082
All ages	Trinidad and Tobago	Alzheimer's disease and other dementias	Rate	0,867592272
All ages	Tunisia	Alzheimer's disease and other dementias	Rate	3,636009249
All ages	Turkey	Alzheimer's disease and other dementias	Rate	2,246087682
All ages	Turkmenistan	Alzheimer's disease and other dementias	Rate	1,012867936
All ages	Tuvalu	Alzheimer's disease and other dementias	Rate	1,78710676
All ages	Uganda	Alzheimer's disease and other dementias	Rate	1,03420482
All ages	Ukraine	Alzheimer's disease and other dementias	Rate	1,247991729
All ages	United Arab Emirates	Alzheimer's disease and other dementias	Rate	3,290514341
All ages	United Kingdom	Alzheimer's disease and other dementias	Rate	2,66074994
All ages	United Republic of Tanzania	Alzheimer's disease and other dementias	Rate	1,560170699
All ages	United States of America	Alzheimer's disease and other dementias	Rate	3,229678288
All ages	United States Virgin Islands	Alzheimer's disease and other dementias	Rate	0,834798877
All ages	Uruguay	Alzheimer's disease and other dementias	Rate	1,367220099
All ages	Uzbekistan	Alzheimer's disease and other dementias	Rate	0,830912988
All ages	Vanuatu	Alzheimer's disease and other dementias	Rate	1,034924763
All ages	Venezuela (Bolivarian Republic of)	Alzheimer's disease and other dementias	Rate	1,104850627
All ages	Viet Nam	Alzheimer's disease and other dementias	Rate	2,263140612
All ages	Yemen	Alzheimer's disease and other dementias	Rate	2,993669507
All ages	Zambia	Alzheimer's disease and other dementias	Rate	1,56230076
All ages	Zimbabwe	Alzheimer's disease and other dementias	Rate	2,284482012

Table S2. Age-standardised DALY rates (ASDR) from smoking-attributable dementia across 204 countries and territories in 2021.

age_name	location_name	cause_name	metric_name	2021-ASDR-Both
All ages	Afghanistan	Alzheimer's disease and other dementias	Rate	32,07597931
All ages	Albania	Alzheimer's disease and other dementias	Rate	91,2313806
All ages	Algeria	Alzheimer's disease and other dementias	Rate	77,82855286
All ages	American Samoa	Alzheimer's disease and other dementias	Rate	36,46671003
All ages	Andorra	Alzheimer's disease and other dementias	Rate	52,36888643
All ages	Angola	Alzheimer's disease and other dementias	Rate	27,7179322
All ages	Antigua and Barbuda	Alzheimer's disease and other dementias	Rate	20,21384806
All ages	Argentina	Alzheimer's disease and other dementias	Rate	35,49989881
All ages	Armenia	Alzheimer's disease and other dementias	Rate	63,82852468
All ages	Australia	Alzheimer's disease and other dementias	Rate	33,23759215
All ages	Austria	Alzheimer's disease and other dementias	Rate	53,98057755
All ages	Azerbaijan	Alzheimer's disease and other dementias	Rate	51,73524454
All ages	Bahamas	Alzheimer's disease and other dementias	Rate	20,49877474
All ages	Bahrain	Alzheimer's disease and other dementias	Rate	54,75357477
All ages	Bangladesh	Alzheimer's disease and other dementias	Rate	45,68869494
All ages	Barbados	Alzheimer's disease and other dementias	Rate	16,8484769
All ages	Belarus	Alzheimer's disease and other dementias	Rate	41,83796755
All ages	Belgium	Alzheimer's disease and other dementias	Rate	61,27260585
All ages	Belize	Alzheimer's disease and other dementias	Rate	25,58295311
All ages	Benin	Alzheimer's disease and other dementias	Rate	7,091436639
All ages	Bermuda	Alzheimer's disease and other dementias	Rate	28,98553152
All ages	Bhutan	Alzheimer's disease and other dementias	Rate	25,35140754
All ages	Bolivia (Plurinational State of)	Alzheimer's disease and other dementias	Rate	16,96212036
All ages	Bosnia and Herzegovina	Alzheimer's disease and other dementias	Rate	67,2862394
All ages	Botswana	Alzheimer's disease and other dementias	Rate	36,56216001
All ages	Brazil	Alzheimer's disease and other dementias	Rate	61,86578657
All ages	Brunei Darussalam	Alzheimer's disease and other dementias	Rate	37,47312179
All ages	Bulgaria	Alzheimer's disease and other dementias	Rate	34,48264364
All ages	Burkina Faso	Alzheimer's disease and other dementias	Rate	6,892614086
All ages	Burundi	Alzheimer's disease and other dementias	Rate	12,05379739
All ages	Cabo Verde	Alzheimer's disease and other dementias	Rate	6,836627802
All ages	Cambodia	Alzheimer's disease and other dementias	Rate	77,84702867
All ages	Cameroon	Alzheimer's disease and other dementias	Rate	9,737351424
All ages	Canada	Alzheimer's disease and other dementias	Rate	59,36087015
All ages	Central African Republic	Alzheimer's disease and other dementias	Rate	14,26185782
All ages	Chad	Alzheimer's disease and other dementias	Rate	12,76266357
All ages	Chile	Alzheimer's disease and other dementias	Rate	21,5929358
All ages	China	Alzheimer's disease and other dementias	Rate	91,49647848
All ages	Colombia	Alzheimer's disease and other dementias	Rate	18,46599834
All ages	Comoros	Alzheimer's disease and other dementias	Rate	25,78415331
All ages	Congo	Alzheimer's disease and other dementias	Rate	26,887382
All ages	Cook Islands	Alzheimer's disease and other dementias	Rate	34,20911588
All ages	Costa Rica	Alzheimer's disease and other dementias	Rate	29,57085958
All ages	Croatia	Alzheimer's disease and other dementias	Rate	66,8425493
All ages	Cuba	Alzheimer's disease and other dementias	Rate	38,14062357
All ages	Cyprus	Alzheimer's disease and other dementias	Rate	59,57782808
All ages	Czechia	Alzheimer's disease and other dementias	Rate	52,48643125
All ages	Côte d'Ivoire	Alzheimer's disease and other dementias	Rate	12,17824376
All ages	Democratic People's Republic of Korea	Alzheimer's disease and other dementias	Rate	36,33343361
All ages	Democratic Republic of the Congo	Alzheimer's disease and other dementias	Rate	10,41094836
All ages	Denmark	Alzheimer's disease and other dementias	Rate	78,82394869
All ages	Djibouti	Alzheimer's disease and other dementias	Rate	39,9195175
All ages	Dominica	Alzheimer's disease and other dementias	Rate	16,04149995
All ages	Dominican Republic	Alzheimer's disease and other dementias	Rate	50,30971231
All ages	Ecuador	Alzheimer's disease and other dementias	Rate	14,91343775
All ages	Egypt	Alzheimer's disease and other dementias	Rate	80,86982387
All ages	El Salvador	Alzheimer's disease and other dementias	Rate	16,99152964
All ages	Equatorial Guinea	Alzheimer's disease and other dementias	Rate	17,21450698
All ages	Eritrea	Alzheimer's disease and other dementias	Rate	7,933812711
All ages	Estonia	Alzheimer's disease and other dementias	Rate	40,39016414
All ages	Eswatini	Alzheimer's disease and other dementias	Rate	21,87266722
All ages	Ethiopia	Alzheimer's disease and other dementias	Rate	9,244747838
All ages	Fiji	Alzheimer's disease and other dementias	Rate	27,23746608
All ages	Finland	Alzheimer's disease and other dementias	Rate	37,34517848
All ages	France	Alzheimer's disease and other dementias	Rate	39,78165565
All ages	Gabon	Alzheimer's disease and other dementias	Rate	16,40376014
All ages	Gambia	Alzheimer's disease and other dementias	Rate	11,82773247
All ages	Georgia	Alzheimer's disease and other dementias	Rate	49,2062256
All ages	Germany	Alzheimer's disease and other dementias	Rate	59,02841837
All ages	Ghana	Alzheimer's disease and other dementias	Rate	13,03287651
All ages	Greece	Alzheimer's disease and other dementias	Rate	72,42870776
All ages	Greenland	Alzheimer's disease and other dementias	Rate	80,44404567
All ages	Grenada	Alzheimer's disease and other dementias	Rate	13,8221998
All ages	Guam	Alzheimer's disease and other dementias	Rate	28,98837555
All ages	Guatemala	Alzheimer's disease and other dementias	Rate	20,88932183
All ages	Guinea	Alzheimer's disease and other dementias	Rate	13,5592261
All ages	Guinea-Bissau	Alzheimer's disease and other dementias	Rate	7,366086571
All ages	Guyana	Alzheimer's disease and other dementias	Rate	17,17210662
All ages	Haiti	Alzheimer's disease and other dementias	Rate	11,7659874
All ages	Honduras	Alzheimer's disease and other dementias	Rate	36,25954211
All ages	Hungary	Alzheimer's disease and other dementias	Rate	37,68659033
All ages	Iceland	Alzheimer's disease and other dementias	Rate	56,99318409
All ages	India	Alzheimer's disease and other dementias	Rate	30,62391727
All ages	Indonesia	Alzheimer's disease and other dementias	Rate	55,04042178
All ages	Iran (Islamic Republic of)	Alzheimer's disease and other dementias	Rate	42,48795162
All ages	Iraq	Alzheimer's disease and other dementias	Rate	75,77138359
All ages	Ireland	Alzheimer's disease and other dementias	Rate	58,18930882
All ages	Israel	Alzheimer's disease and other dementias	Rate	43,54298114
All ages	Italy	Alzheimer's disease and other dementias	Rate	51,99030017
All ages	Jamaica	Alzheimer's disease and other dementias	Rate	26,48282184
All ages	Japan	Alzheimer's disease and other dementias	Rate	45,92846596
All ages	Jordan	Alzheimer's disease and other dementias	Rate	84,35627861
All ages	Kazakhstan	Alzheimer's disease and other dementias	Rate	23,26888433
All ages	Kenya	Alzheimer's disease and other dementias	Rate	18,0937149
All ages	Kiribati	Alzheimer's disease and other dementias	Rate	84,26431738
All ages	Kuwait	Alzheimer's disease and other dementias	Rate	65,95163949
All ages	Kyrgyzstan	Alzheimer's disease and other dementias	Rate	55,86031999
All ages	Lao People's Democratic Republic	Alzheimer's disease and other dementias	Rate	59,90683932
All ages	Latvia	Alzheimer's disease and other dementias	Rate	31,20540211
All ages	Lebanon	Alzheimer's disease and other dementias	Rate	106,4404824
All ages	Lesotho	Alzheimer's disease and other dementias	Rate	49,89217222
All ages	Liberia	Alzheimer's disease and other dementias	Rate	7,81755992
All ages	Libya	Alzheimer's disease and other dementias	Rate	52,91100427
All ages	Lithuania	Alzheimer's disease and other dementias	Rate	32,72096059
All ages	Luxembourg	Alzheimer's disease and other dementias	Rate	38,86257044
All ages	Madagascar	Alzheimer's disease and other dementias	Rate	10,81316416
All ages	Malawi	Alzheimer's disease and other dementias	Rate	30,77043252
All ages	Malaysia	Alzheimer's disease and other dementias	Rate	45,66589623
All ages	Maldives	Alzheimer's disease and other dementias	Rate	64,86395795
All ages	Mali	Alzheimer's disease and other dementias	Rate	21,04478707
All ages	Malta	Alzheimer's disease and other dementias	Rate	39,2488608
All ages	Marshall Islands	Alzheimer's disease and other dementias	Rate	31,95347259
All ages	Mauritania	Alzheimer's disease and other dementias	Rate	9,155471796
All ages	Mauritius	Alzheimer's disease and other dementias	Rate	42,68744278
All ages	Mexico	Alzheimer's disease and other dementias	Rate	18,15320891
All ages	Micronesia (Federated States of)	Alzheimer's disease and other dementias	Rate	47,35079921
All ages	Monaco	Alzheimer's disease and other dementias	Rate	54,97013663
All ages	Mongolia	Alzheimer's disease and other dementias	Rate	43,98625551
All ages	Montenegro	Alzheimer's disease and other dementias	Rate	60,66025735
All ages	Morocco	Alzheimer's disease and other dementias	Rate	23,66447892
All ages	Mozambique	Alzheimer's disease and other dementias	Rate	21,02825449
All ages	Myanmar	Alzheimer's disease and other dementias	Rate	44,60746426
All ages	Namibia	Alzheimer's disease and other dementias	Rate	46,84110546
All ages	Nauru	Alzheimer's disease and other dementias	Rate	41,99890209
All ages	Nepal	Alzheimer's disease and other dementias	Rate	56,32987352
All ages	Netherlands	Alzheimer's disease and other dementias	Rate	71,81598022
All ages	New Zealand	Alzheimer's disease and other dementias	Rate	52,43993596
All ages	Nicaragua	Alzheimer's disease and other dementias	Rate	24,89721772
All ages	Niger	Alzheimer's disease and other dementias	Rate	8,720112132
All ages	Nigeria	Alzheimer's disease and other dementias	Rate	6,105714851
All ages	Niue	Alzheimer's disease and other dementias	Rate	31,46456642
All ages	North Macedonia	Alzheimer's disease and other dementias	Rate	62,62577207
All ages	Northern Mariana Islands	Alzheimer's disease and other dementias	Rate	34,75577118
All ages	Norway	Alzheimer's disease and other dementias	Rate	41,43082987
All ages	Oman	Alzheimer's disease and other dementias	Rate	25,71870714
All ages	Pakistan	Alzheimer's disease and other dementias	Rate	33,31884503
All ages	Palau	Alzheimer's disease and other dementias	Rate	32,1330694
All ages	Palestine	Alzheimer's disease and other dementias	Rate	60,4727157
All ages	Panama	Alzheimer's disease and other dementias	Rate	24,08374419
All ages	Papua New Guinea	Alzheimer's disease and other dementias	Rate	28,31312287
All ages	Paraguay	Alzheimer's disease and other dementias	Rate	75,98210079
All ages	Peru	Alzheimer's disease and other dementias	Rate	17,58002043
All ages	Philippines	Alzheimer's disease and other dementias	Rate	50,28627295
All ages	Poland	Alzheimer's disease and other dementias	Rate	44,60767935
All ages	Portugal	Alzheimer's disease and other dementias	Rate	23,64968712
All ages	Puerto Rico	Alzheimer's disease and other dementias	Rate	27,24331311
All ages	Qatar	Alzheimer's disease and other dementias	Rate	34,66550129
All ages	Republic of Korea	Alzheimer's disease and other dementias	Rate	62,67156393
All ages	Republic of Moldova	Alzheimer's disease and other dementias	Rate	35,44349809
All ages	Romania	Alzheimer's disease and other dementias	Rate	30,67639223
All ages	Russian Federation	Alzheimer's disease and other dementias	Rate	36,75954794
All ages	Rwanda	Alzheimer's disease and other dementias	Rate	78,46148997
All ages	Saint Kitts and Nevis	Alzheimer's disease and other dementias	Rate	13,49218299
All ages	Saint Lucia	Alzheimer's disease and other dementias	Rate	17,92969322
All ages	Saint Vincent and the Grenadines	Alzheimer's disease and other dementias	Rate	18,30922411
All ages	Samoa	Alzheimer's disease and other dementias	Rate	53,24059629
All ages	San Marino	Alzheimer's disease and other dementias	Rate	47,43979491
All ages	Sao Tome and Principe	Alzheimer's disease and other dementias	Rate	6,491102456
All ages	Saudi Arabia	Alzheimer's disease and other dementias	Rate	28,78370496
All ages	Senegal	Alzheimer's disease and other dementias	Rate	9,365654379
All ages	Serbia	Alzheimer's disease and other dementias	Rate	47,13848194
All ages	Seychelles	Alzheimer's disease and other dementias	Rate	45,21182521
All ages	Sierra Leone	Alzheimer's disease and other dementias	Rate	9,874416829
All ages	Singapore	Alzheimer's disease and other dementias	Rate	19,73103106
All ages	Slovakia	Alzheimer's disease and other dementias	Rate	37,32054935
All ages	Slovenia	Alzheimer's disease and other dementias	Rate	41,00582982
All ages	Solomon Islands	Alzheimer's disease and other dementias	Rate	51,77961536
All ages	Somalia	Alzheimer's disease and other dementias	Rate	17,31415732
All ages	South Africa	Alzheimer's disease and other dementias	Rate	22,11857067
All ages	South Sudan	Alzheimer's disease and other dementias	Rate	20,78684298
All ages	Spain	Alzheimer's disease and other dementias	Rate	37,69052775
All ages	Sri Lanka	Alzheimer's disease and other dementias	Rate	21,74099869
All ages	Sudan	Alzheimer's disease and other dementias	Rate	46,08340132
All ages	Suriname	Alzheimer's disease and other dementias	Rate	26,58643633
All ages	Sweden	Alzheimer's disease and other dementias	Rate	66,43440106
All ages	Switzerland	Alzheimer's disease and other dementias	Rate	59,85085328
All ages	Syrian Arab Republic	Alzheimer's disease and other dementias	Rate	60,30951525
All ages	Taiwan (Province of China)	Alzheimer's disease and other dementias	Rate	36,16791611
All ages	Tajikistan	Alzheimer's disease and other dementias	Rate	28,59498735
All ages	Thailand	Alzheimer's disease and other dementias	Rate	42,00117533
All ages	Timor-Leste	Alzheimer's disease and other dementias	Rate	49,83654862
All ages	Togo	Alzheimer's disease and other dementias	Rate	17,3629835
All ages	Tokelau	Alzheimer's disease and other dementias	Rate	37,88443815
All ages	Tonga	Alzheimer's disease and other dementias	Rate	60,27130299
All ages	Trinidad and Tobago	Alzheimer's disease and other dementias	Rate	22,55923862
All ages	Tunisia	Alzheimer's disease and other dementias	Rate	74,70817967
All ages	Turkey	Alzheimer's disease and other dementias	Rate	51,79645092
All ages	Turkmenistan	Alzheimer's disease and other dementias	Rate	25,11665352
All ages	Tuvalu	Alzheimer's disease and other dementias	Rate	42,60809837
All ages	Uganda	Alzheimer's disease and other dementias	Rate	19,51841135
All ages	Ukraine	Alzheimer's disease and other dementias	Rate	31,0275025
All ages	United Arab Emirates	Alzheimer's disease and other dementias	Rate	62,3920743
All ages	United Kingdom	Alzheimer's disease and other dementias	Rate	54,31982156
All ages	United Republic of Tanzania	Alzheimer's disease and other dementias	Rate	31,15089058
All ages	United States of America	Alzheimer's disease and other dementias	Rate	70,94463874
All ages	United States Virgin Islands	Alzheimer's disease and other dementias	Rate	21,2827786
All ages	Uruguay	Alzheimer's disease and other dementias	Rate	36,27817129
All ages	Uzbekistan	Alzheimer's disease and other dementias	Rate	21,00051497
All ages	Vanuatu	Alzheimer's disease and other dementias	Rate	24,35876501
All ages	Venezuela (Bolivarian Republic of)	Alzheimer's disease and other dementias	Rate	27,15400136
All ages	Viet Nam	Alzheimer's disease and other dementias	Rate	50,82539036
All ages	Yemen	Alzheimer's disease and other dementias	Rate	65,13229316
All ages	Zambia	Alzheimer's disease and other dementias	Rate	29,99952573
All ages	Zimbabwe	Alzheimer's disease and other dementias	Rate	45,06532734

Table S2. Estimated annual percentage change (EAPC) in age-standardised mortality rates (ASMR) from smoking-attributable dementia across 204 countries and territories from 1990 to 2021.

location_name	EAPC-ASMR	EAPCs
Afghanistan	0,172553322	0.17(-0.08,0.42)
Albania	0,611505394	0.61(0.52,0.70)
Algeria	-0,81012795	-0.81(-1.05,-0.57)
American Samoa	-0,54201986	-0.54(-0.62,-0.47)
Andorra	-1,022687261	-1.02(-1.07,-0.98)
Angola	-0,045778016	-0.05(-0.28,0.19)
Antigua and Barbuda	-0,328872701	-0.33(-0.38,-0.28)
Argentina	-0,174153844	-0.17(-0.30,-0.04)
Armenia	1,18476498	1.18(1.00,1.37)
Australia	-1,187477339	-1.19(-1.32,-1.05)
Austria	0,704364289	0.70(0.38,1.03)
Azerbaijan	1,822809785	1.82(1.61,2.04)
Bahamas	0,162312688	0.16(0.09,0.24)
Bahrain	-0,893171376	-0.89(-1.01,-0.78)
Bangladesh	-0,218176874	-0.22(-0.38,-0.06)
Barbados	-0,728053954	-0.73(-0.99,-0.46)
Belarus	-0,227288781	-0.23(-0.37,-0.09)
Belgium	-1,247452825	-1.25(-1.44,-1.06)
Belize	-0,251580908	-0.25(-0.34,-0.16)
Benin	-1,990254635	-1.99(-2.18,-1.80)
Bermuda	0,302474524	0.30(0.26,0.34)
Bhutan	0,335291155	0.34(0.30,0.37)
Bolivia (Plurinational State of)	-0,415861579	-0.42(-0.55,-0.28)
Bosnia and Herzegovina	0,187855099	0.19(-0.18,0.56)
Botswana	-1,132503884	-1.13(-1.23,-1.03)
Brazil	-2,031057004	-2.03(-2.22,-1.84)
Brunei Darussalam	-1,678767154	-1.68(-1.76,-1.60)
Bulgaria	-1,520823292	-1.52(-1.61,-1.43)
Burkina Faso	-0,769696984	-0.77(-1.03,-0.51)
Burundi	-1,784243362	-1.78(-2.15,-1.42)
Cabo Verde	-2,141179317	-2.14(-2.41,-1.87)
Cambodia	0,260729257	0.26(0.07,0.45)
Cameroon	-1,054860158	-1.05(-1.12,-0.99)
Canada	-1,630373973	-1.63(-1.74,-1.52)
Central African Republic	-1,313130537	-1.31(-1.56,-1.07)
Chad	-0,49192137	-0.49(-0.56,-0.43)
Chile	-1,024832398	-1.02(-1.10,-0.94)
China	-0,740905696	-0.74(-0.83,-0.65)
Colombia	-1,577978685	-1.58(-1.68,-1.47)
Comoros	-0,343514838	-0.34(-0.38,-0.31)
Congo	0,900922808	0.90(0.68,1.12)
Cook Islands	-0,95702294	-0.96(-1.00,-0.91)
Costa Rica	-1,281311291	-1.28(-1.50,-1.06)
Croatia	-0,127363127	-0.13(-0.29,0.04)
Cuba	-1,461486549	-1.46(-1.57,-1.35)
Cyprus	-1,107427731	-1.11(-1.20,-1.02)
Czechia	-0,601486046	-0.60(-0.64,-0.57)
Côte d'Ivoire	-1,457437594	-1.46(-1.69,-1.22)
Democratic People's Republic of Korea	-0,892661571	-0.89(-0.97,-0.82)
Democratic Republic of the Congo	-1,068403803	-1.07(-1.36,-0.77)
Denmark	-1,831976712	-1.83(-2.03,-1.63)
Djibouti	-0,109227422	-0.11(-0.17,-0.05)
Dominica	-0,501248182	-0.50(-0.67,-0.33)
Dominican Republic	-0,848074277	-0.85(-1.10,-0.60)
Ecuador	-2,269209753	-2.27(-2.35,-2.19)
Egypt	0,284242807	0.28(0.18,0.39)
El Salvador	0,062075277	0.06(0.01,0.12)
Equatorial Guinea	-0,262932999	-0.26(-0.33,-0.20)
Eritrea	-0,36671371	-0.37(-0.51,-0.23)
Estonia	0,635729058	0.64(0.38,0.90)
Eswatini	-1,244794077	-1.24(-1.32,-1.17)
Ethiopia	0,652965675	0.65(0.39,0.92)
Fiji	-1,095302826	-1.10(-1.27,-0.92)
Finland	-0,133160629	-0.13(-0.34,0.07)
France	-1,34842903	-1.35(-1.44,-1.26)
Gabon	0,012370751	0.01(-0.10,0.12)
Gambia	-1,783893925	-1.78(-1.89,-1.68)
Georgia	1,743381538	1.74(1.60,1.89)
Germany	-1,042219385	-1.04(-1.22,-0.86)
Ghana	0,541439674	0.54(0.27,0.81)
Greece	-1,130340962	-1.13(-1.19,-1.08)
Greenland	-0,710650077	-0.71(-0.75,-0.67)
Grenada	-1,475402819	-1.48(-1.66,-1.29)
Guam	-0,232645173	-0.23(-0.29,-0.18)
Guatemala	-0,954092548	-0.95(-1.09,-0.82)
Guinea	-0,619247259	-0.62(-0.67,-0.57)
Guinea-Bissau	0,45209391	0.45(0.17,0.73)
Guyana	-0,531928811	-0.53(-0.63,-0.43)
Haiti	-0,463388664	-0.46(-0.56,-0.36)
Honduras	0,127889965	0.13(0.05,0.20)
Hungary	-1,196144287	-1.20(-1.23,-1.16)
Iceland	-2,035930139	-2.04(-2.13,-1.94)
India	-0,461043024	-0.46(-0.59,-0.34)
Indonesia	0,718817238	0.72(0.45,0.99)
Iran (Islamic Republic of)	0,209142632	0.21(0.13,0.29)
Iraq	-1,341361574	-1.34(-1.39,-1.29)
Ireland	-2,819622361	-2.82(-2.92,-2.71)
Israel	-1,495189119	-1.50(-1.62,-1.37)
Italy	-1,780712101	-1.78(-1.83,-1.73)
Jamaica	-1,024785082	-1.02(-1.12,-0.93)
Japan	-1,832826673	-1.83(-1.92,-1.75)
Jordan	-1,25429515	-1.25(-1.39,-1.11)
Kazakhstan	-0,28112415	-0.28(-0.42,-0.15)
Kenya	-2,338832938	-2.34(-2.52,-2.16)
Kiribati	0,388898939	0.39(0.07,0.70)
Kuwait	-0,26394046	-0.26(-0.40,-0.13)
Kyrgyzstan	2,291530553	2.29(1.97,2.62)
Lao People's Democratic Republic	0,521330945	0.52(0.44,0.60)
Latvia	-0,675525662	-0.68(-0.76,-0.59)
Lebanon	0,543884859	0.54(0.24,0.85)
Lesotho	0,722712456	0.72(0.61,0.83)
Liberia	-1,350810694	-1.35(-1.43,-1.27)
Libya	-0,597453125	-0.60(-0.69,-0.51)
Lithuania	-1,152975931	-1.15(-1.21,-1.10)
Luxembourg	-0,300516844	-0.30(-0.42,-0.18)
Madagascar	-2,85244879	-2.85(-3.23,-2.47)
Malawi	-0,713480599	-0.71(-0.76,-0.66)
Malaysia	-1,334230547	-1.33(-1.46,-1.21)
Maldives	-1,562601615	-1.56(-1.62,-1.50)
Mali	2,031404161	2.03(1.88,2.18)
Malta	-1,807303328	-1.81(-1.93,-1.68)
Marshall Islands	0,171082285	0.17(0.15,0.19)
Mauritania	-0,582905999	-0.58(-0.63,-0.53)
Mauritius	-0,118741269	-0.12(-0.54,0.30)
Mexico	-3,295734331	-3.30(-3.51,-3.08)
Micronesia (Federated States of)	-0,475206454	-0.48(-0.51,-0.44)
Monaco	-0,513528287	-0.51(-0.55,-0.48)
Mongolia	1,133985011	1.13(0.94,1.33)
Montenegro	-0,539470755	-0.54(-0.71,-0.36)
Morocco	-2,056638623	-2.06(-2.14,-1.97)
Mozambique	-0,674011335	-0.67(-0.80,-0.55)
Myanmar	-2,244325374	-2.24(-2.36,-2.13)
Namibia	-1,206997797	-1.21(-1.36,-1.05)
Nauru	-1,184956142	-1.18(-1.23,-1.14)
Nepal	-0,664587783	-0.66(-0.76,-0.57)
Netherlands	-0,44444665	-0.44(-0.58,-0.31)
New Zealand	-0,635085676	-0.64(-0.73,-0.54)
Nicaragua	0,001563079	0.00(-0.19,0.19)
Niger	0,489015496	0.49(0.29,0.69)
Nigeria	-1,6214631	-1.62(-1.66,-1.58)
Niue	-0,32203476	-0.32(-0.36,-0.28)
North Macedonia	-0,455208725	-0.46(-0.64,-0.27)
Northern Mariana Islands	-0,824145991	-0.82(-0.87,-0.78)
Norway	-2,588577221	-2.59(-2.86,-2.32)
Oman	-0,831428341	-0.83(-0.96,-0.71)
Pakistan	-1,614416728	-1.61(-1.77,-1.46)
Palau	-0,38041465	-0.38(-0.42,-0.34)
Palestine	-1,280976294	-1.28(-1.41,-1.15)
Panama	-1,768467904	-1.77(-1.91,-1.63)
Papua New Guinea	-0,648344534	-0.65(-0.72,-0.57)
Paraguay	-1,460047666	-1.46(-1.54,-1.38)
Peru	-0,315364363	-0.32(-0.47,-0.16)
Philippines	-1,793979616	-1.79(-1.92,-1.67)
Poland	-1,425550445	-1.43(-1.50,-1.35)
Portugal	-1,802744001	-1.80(-1.91,-1.69)
Puerto Rico	-0,808045117	-0.81(-0.87,-0.75)
Qatar	-1,157425516	-1.16(-1.30,-1.02)
Republic of Korea	-1,910764188	-1.91(-1.99,-1.83)
Republic of Moldova	0,539487601	0.54(0.45,0.63)
Romania	-1,664287737	-1.66(-1.86,-1.47)
Russian Federation	1,137571098	1.14(0.75,1.53)
Rwanda	0,905877497	0.91(0.81,1.00)
Saint Kitts and Nevis	-0,665727995	-0.67(-0.74,-0.59)
Saint Lucia	-1,106838076	-1.11(-1.23,-0.98)
Saint Vincent and the Grenadines	0,061537372	0.06(-0.00,0.13)
Samoa	-0,365862214	-0.37(-0.43,-0.30)
San Marino	-0,856279898	-0.86(-0.92,-0.80)
Sao Tome and Principe	-0,231374106	-0.23(-0.57,0.11)
Saudi Arabia	-0,324845484	-0.32(-0.35,-0.30)
Senegal	-0,656299182	-0.66(-0.81,-0.50)
Serbia	-0,549046677	-0.55(-0.91,-0.19)
Seychelles	-0,815826031	-0.82(-0.91,-0.72)
Sierra Leone	-1,117724584	-1.12(-1.20,-1.04)
Singapore	-1,675893698	-1.68(-1.79,-1.56)
Slovakia	-0,687964225	-0.69(-0.83,-0.54)
Slovenia	-0,014316271	-0.01(-0.19,0.16)
Solomon Islands	-0,179745522	-0.18(-0.36,-0.00)
Somalia	-0,922552949	-0.92(-0.99,-0.85)
South Africa	-3,436664108	-3.44(-3.55,-3.32)
South Sudan	-0,420062604	-0.42(-0.46,-0.38)
Spain	-2,152463384	-2.15(-2.19,-2.11)
Sri Lanka	-2,977347885	-2.98(-3.28,-2.68)
Sudan	-0,285325483	-0.29(-0.31,-0.26)
Suriname	-1,699691717	-1.70(-1.88,-1.52)
Sweden	-0,197504388	-0.20(-0.35,-0.05)
Switzerland	-0,465993956	-0.47(-0.53,-0.40)
Syrian Arab Republic	-1,172829475	-1.17(-1.27,-1.08)
Taiwan (Province of China)	-1,296051671	-1.30(-1.40,-1.19)
Tajikistan	-1,350841426	-1.35(-1.56,-1.14)
Thailand	-1,737103104	-1.74(-1.87,-1.61)
Timor-Leste	-0,406986806	-0.41(-0.52,-0.30)
Togo	-1,736072215	-1.74(-1.88,-1.60)
Tokelau	-0,916164974	-0.92(-0.98,-0.85)
Tonga	-0,77894532	-0.78(-0.88,-0.68)
Trinidad and Tobago	-0,949245153	-0.95(-1.00,-0.89)
Tunisia	-1,114078835	-1.11(-1.20,-1.03)
Turkey	-2,099375228	-2.10(-2.40,-1.79)
Turkmenistan	-1,382246046	-1.38(-1.60,-1.16)
Tuvalu	-0,086871619	-0.09(-0.13,-0.04)
Uganda	-0,821033993	-0.82(-1.07,-0.57)
Ukraine	-0,811093686	-0.81(-1.05,-0.57)
United Arab Emirates	1,17671442	1.18(0.99,1.36)
United Kingdom	-1,864529773	-1.86(-1.98,-1.75)
United Republic of Tanzania	-1,620043389	-1.62(-1.75,-1.49)
United States of America	-1,197914039	-1.20(-1.27,-1.13)
United States Virgin Islands	-0,520758426	-0.52(-0.61,-0.43)
Uruguay	0,239913895	0.24(0.15,0.33)
Uzbekistan	0,714816408	0.71(0.51,0.92)
Vanuatu	-1,207528094	-1.21(-1.33,-1.09)
Venezuela (Bolivarian Republic of)	-1,940877575	-1.94(-1.99,-1.89)
Viet Nam	-1,374870213	-1.37(-1.59,-1.16)
Yemen	-0,047714674	-0.05(-0.08,-0.02)
Zambia	-0,796509771	-0.80(-0.87,-0.72)
Zimbabwe	-0,745884023	-0.75(-0.95,-0.54)

Table S2. Estimated annual percentage change (EAPC) in age-standardised DALY rates (ASDR) from smoking-attributable dementia across 204 countries and territories from 1990 to 2021.

location_name	EAPC-ASDR	EAPCs
Afghanistan	0,123210665	0.12(-0.14,0.38)
Albania	0,516807062	0.52(0.45,0.59)
Algeria	-0,878450445	-0.88(-1.05,-0.70)
American Samoa	-0,46310034	-0.46(-0.52,-0.40)
Andorra	-0,981857368	-0.98(-1.03,-0.94)
Angola	-0,126398859	-0.13(-0.35,0.10)
Antigua and Barbuda	-0,318921652	-0.32(-0.38,-0.26)
Argentina	-0,372133148	-0.37(-0.49,-0.26)
Armenia	0,909263321	0.91(0.78,1.04)
Australia	-1,486377089	-1.49(-1.58,-1.39)
Austria	0,644740978	0.64(0.37,0.92)
Azerbaijan	1,399291656	1.40(1.17,1.63)
Bahamas	0,04035764	0.04(-0.02,0.10)
Bahrain	-0,858753014	-0.86(-0.92,-0.80)
Bangladesh	-0,26443397	-0.26(-0.44,-0.09)
Barbados	-0,886031846	-0.89(-1.10,-0.67)
Belarus	0,015628112	0.02(-0.10,0.13)
Belgium	-1,289639621	-1.29(-1.42,-1.15)
Belize	-0,345043686	-0.35(-0.44,-0.25)
Benin	-1,928044826	-1.93(-2.11,-1.75)
Bermuda	0,229794783	0.23(0.20,0.26)
Bhutan	-0,032122593	-0.03(-0.05,-0.02)
Bolivia (Plurinational State of)	-0,460342655	-0.46(-0.62,-0.30)
Bosnia and Herzegovina	0,218495578	0.22(-0.15,0.59)
Botswana	-1,082748885	-1.08(-1.18,-0.98)
Brazil	-1,867691671	-1.87(-2.06,-1.67)
Brunei Darussalam	-1,759492847	-1.76(-1.84,-1.68)
Bulgaria	-1,2402841	-1.24(-1.32,-1.16)
Burkina Faso	-0,762961035	-0.76(-1.02,-0.51)
Burundi	-1,675363656	-1.68(-2.07,-1.28)
Cabo Verde	-2,012744231	-2.01(-2.24,-1.78)
Cambodia	-0,035734985	-0.04(-0.24,0.17)
Cameroon	-1,076654626	-1.08(-1.13,-1.02)
Canada	-1,796954806	-1.80(-1.90,-1.70)
Central African Republic	-1,290483145	-1.29(-1.55,-1.03)
Chad	-0,637934123	-0.64(-0.71,-0.57)
Chile	-1,003304432	-1.00(-1.08,-0.93)
China	-0,485428464	-0.49(-0.58,-0.39)
Colombia	-1,567611085	-1.57(-1.68,-1.46)
Comoros	-0,702096559	-0.70(-0.74,-0.66)
Congo	0,875245037	0.88(0.65,1.11)
Cook Islands	-0,87803951	-0.88(-0.93,-0.83)
Costa Rica	-1,249692462	-1.25(-1.43,-1.07)
Croatia	-0,146067821	-0.15(-0.30,0.01)
Cuba	-1,403357318	-1.40(-1.54,-1.27)
Cyprus	-0,770553649	-0.77(-0.81,-0.73)
Czechia	-0,518607507	-0.52(-0.54,-0.50)
Côte d'Ivoire	-1,363933153	-1.36(-1.59,-1.13)
Democratic People's Republic of Korea	-0,735124921	-0.74(-0.79,-0.68)
Democratic Republic of the Congo	-0,876215628	-0.88(-1.16,-0.60)
Denmark	-1,893350267	-1.89(-2.06,-1.72)
Djibouti	-0,244810827	-0.24(-0.31,-0.18)
Dominica	-0,418884968	-0.42(-0.61,-0.23)
Dominican Republic	-0,860706749	-0.86(-1.09,-0.63)
Ecuador	-2,179297908	-2.18(-2.27,-2.09)
Egypt	0,186373621	0.19(0.10,0.28)
El Salvador	0,200675506	0.20(0.12,0.28)
Equatorial Guinea	-0,376372618	-0.38(-0.44,-0.31)
Eritrea	-0,612488541	-0.61(-0.76,-0.47)
Estonia	0,554577965	0.55(0.31,0.80)
Eswatini	-1,359902654	-1.36(-1.44,-1.28)
Ethiopia	0,095708817	0.10(-0.13,0.33)
Fiji	-1,056587009	-1.06(-1.20,-0.91)
Finland	-0,187882451	-0.19(-0.37,-0.00)
France	-1,10544989	-1.11(-1.14,-1.07)
Gabon	0,17089701	0.17(0.11,0.23)
Gambia	-1,960115777	-1.96(-2.08,-1.84)
Georgia	1,492901246	1.49(1.35,1.63)
Germany	-0,881337047	-0.88(-1.02,-0.74)
Ghana	0,519803107	0.52(0.24,0.80)
Greece	-1,056078375	-1.06(-1.10,-1.02)
Greenland	-0,715891126	-0.72(-0.74,-0.69)
Grenada	-1,23947174	-1.24(-1.38,-1.10)
Guam	-0,064497763	-0.06(-0.10,-0.03)
Guatemala	-0,90564732	-0.91(-1.03,-0.78)
Guinea	-0,557069385	-0.56(-0.61,-0.51)
Guinea-Bissau	0,551909551	0.55(0.26,0.84)
Guyana	-0,535807122	-0.54(-0.62,-0.45)
Haiti	-0,774549707	-0.77(-0.90,-0.65)
Honduras	-0,160406325	-0.16(-0.21,-0.11)
Hungary	-1,05728784	-1.06(-1.09,-1.03)
Iceland	-1,970986675	-1.97(-2.05,-1.89)
India	-0,759552631	-0.76(-0.83,-0.69)
Indonesia	0,397486482	0.40(0.20,0.60)
Iran (Islamic Republic of)	0,036415432	0.04(-0.03,0.10)
Iraq	-1,350881215	-1.35(-1.39,-1.31)
Ireland	-2,701684895	-2.70(-2.80,-2.60)
Israel	-1,481868393	-1.48(-1.57,-1.39)
Italy	-1,476906264	-1.48(-1.51,-1.44)
Jamaica	-1,121039734	-1.12(-1.21,-1.03)
Japan	-1,576809516	-1.58(-1.64,-1.52)
Jordan	-1,082399848	-1.08(-1.18,-0.98)
Kazakhstan	-0,202466013	-0.20(-0.35,-0.06)
Kenya	-2,196772365	-2.20(-2.37,-2.02)
Kiribati	0,251088986	0.25(-0.05,0.56)
Kuwait	-0,514600899	-0.51(-0.59,-0.44)
Kyrgyzstan	2,282433136	2.28(1.98,2.59)
Lao People's Democratic Republic	0,218184295	0.22(0.17,0.26)
Latvia	-0,350689168	-0.35(-0.43,-0.27)
Lebanon	0,407756183	0.41(0.12,0.69)
Lesotho	0,60734808	0.61(0.52,0.69)
Liberia	-1,149710262	-1.15(-1.21,-1.09)
Libya	-0,647942934	-0.65(-0.73,-0.57)
Lithuania	-0,699311238	-0.70(-0.72,-0.67)
Luxembourg	-0,550712007	-0.55(-0.64,-0.46)
Madagascar	-2,815419212	-2.82(-3.19,-2.44)
Malawi	-0,50533278	-0.51(-0.54,-0.47)
Malaysia	-1,38036148	-1.38(-1.51,-1.25)
Maldives	-1,529598601	-1.53(-1.63,-1.43)
Mali	1,87845653	1.88(1.74,2.01)
Malta	-1,665923188	-1.67(-1.75,-1.58)
Marshall Islands	0,113776652	0.11(0.10,0.13)
Mauritania	-0,731683501	-0.73(-0.78,-0.68)
Mauritius	-0,13889737	-0.14(-0.53,0.25)
Mexico	-3,135873559	-3.14(-3.32,-2.95)
Micronesia (Federated States of)	-0,371382223	-0.37(-0.40,-0.34)
Monaco	-0,742935332	-0.74(-0.77,-0.71)
Mongolia	0,972469154	0.97(0.84,1.10)
Montenegro	-0,527845247	-0.53(-0.75,-0.31)
Morocco	-1,791593967	-1.79(-1.91,-1.67)
Mozambique	-0,621277049	-0.62(-0.74,-0.50)
Myanmar	-2,483517339	-2.48(-2.60,-2.37)
Namibia	-1,285591377	-1.29(-1.41,-1.16)
Nauru	-0,954713966	-0.95(-1.00,-0.91)
Nepal	-1,161360108	-1.16(-1.26,-1.07)
Netherlands	-0,453714595	-0.45(-0.59,-0.32)
New Zealand	-0,976357699	-0.98(-1.07,-0.88)
Nicaragua	-0,128978042	-0.13(-0.30,0.04)
Niger	0,063660151	0.06(-0.07,0.20)
Nigeria	-1,350616799	-1.35(-1.43,-1.27)
Niue	-0,38423925	-0.38(-0.43,-0.34)
North Macedonia	-0,527698811	-0.53(-0.64,-0.42)
Northern Mariana Islands	-0,740246281	-0.74(-0.79,-0.69)
Norway	-2,699296683	-2.70(-2.95,-2.45)
Oman	-0,917466386	-0.92(-1.03,-0.80)
Pakistan	-1,734672547	-1.73(-1.87,-1.60)
Palau	-0,451392364	-0.45(-0.50,-0.40)
Palestine	-1,002971393	-1.00(-1.11,-0.90)
Panama	-1,689442783	-1.69(-1.82,-1.56)
Papua New Guinea	-0,61106158	-0.61(-0.68,-0.54)
Paraguay	-1,508298665	-1.51(-1.58,-1.44)
Peru	-0,255449024	-0.26(-0.41,-0.11)
Philippines	-1,738091987	-1.74(-1.87,-1.61)
Poland	-1,315060166	-1.32(-1.34,-1.29)
Portugal	-1,630554315	-1.63(-1.71,-1.55)
Puerto Rico	-0,664775646	-0.66(-0.71,-0.62)
Qatar	-0,742833699	-0.74(-0.82,-0.67)
Republic of Korea	-1,682223009	-1.68(-1.76,-1.60)
Republic of Moldova	0,599405263	0.60(0.51,0.68)
Romania	-1,284316813	-1.28(-1.43,-1.14)
Russian Federation	0,986882232	0.99(0.63,1.35)
Rwanda	0,730534949	0.73(0.64,0.82)
Saint Kitts and Nevis	-0,816298594	-0.82(-0.91,-0.72)
Saint Lucia	-1,191910237	-1.19(-1.35,-1.04)
Saint Vincent and the Grenadines	0,041899912	0.04(-0.00,0.09)
Samoa	-0,457147812	-0.46(-0.53,-0.39)
San Marino	-1,030468423	-1.03(-1.08,-0.98)
Sao Tome and Principe	-0,530231844	-0.53(-0.81,-0.25)
Saudi Arabia	-0,153218149	-0.15(-0.19,-0.12)
Senegal	-0,908716022	-0.91(-1.05,-0.77)
Serbia	-0,351674421	-0.35(-0.67,-0.03)
Seychelles	-0,745873588	-0.75(-0.83,-0.66)
Sierra Leone	-1,033417349	-1.03(-1.10,-0.97)
Singapore	-1,4211525	-1.42(-1.52,-1.32)
Slovakia	-0,40641422	-0.41(-0.57,-0.24)
Slovenia	0,173766816	0.17(-0.00,0.35)
Solomon Islands	-0,041590797	-0.04(-0.24,0.16)
Somalia	-0,906629409	-0.91(-1.00,-0.81)
South Africa	-3,1414329	-3.14(-3.28,-3.01)
South Sudan	-0,684050504	-0.68(-0.75,-0.61)
Spain	-1,858703764	-1.86(-1.88,-1.83)
Sri Lanka	-2,841607275	-2.84(-3.13,-2.56)
Sudan	-0,43414528	-0.43(-0.48,-0.39)
Suriname	-1,729442284	-1.73(-1.90,-1.56)
Sweden	-0,199641355	-0.20(-0.31,-0.09)
Switzerland	-0,671769409	-0.67(-0.73,-0.61)
Syrian Arab Republic	-1,334248538	-1.33(-1.40,-1.26)
Taiwan (Province of China)	-1,457341572	-1.46(-1.54,-1.38)
Tajikistan	-1,40293302	-1.40(-1.54,-1.27)
Thailand	-1,604993527	-1.60(-1.69,-1.52)
Timor-Leste	-0,649058725	-0.65(-0.71,-0.58)
Togo	-1,568492882	-1.57(-1.72,-1.42)
Tokelau	-0,82741872	-0.83(-0.89,-0.77)
Tonga	-0,79716865	-0.80(-0.88,-0.72)
Trinidad and Tobago	-1,000193936	-1.00(-1.05,-0.95)
Tunisia	-1,131971477	-1.13(-1.17,-1.09)
Turkey	-2,062788063	-2.06(-2.27,-1.86)
Turkmenistan	-1,213005402	-1.21(-1.32,-1.10)
Tuvalu	-0,150906674	-0.15(-0.19,-0.11)
Uganda	-0,832750754	-0.83(-1.06,-0.61)
Ukraine	-0,712452473	-0.71(-0.97,-0.45)
United Arab Emirates	0,835801875	0.84(0.70,0.97)
United Kingdom	-1,947516538	-1.95(-2.06,-1.84)
United Republic of Tanzania	-1,510347694	-1.51(-1.63,-1.39)
United States of America	-1,356228967	-1.36(-1.42,-1.29)
United States Virgin Islands	-0,446057887	-0.45(-0.50,-0.39)
Uruguay	-0,027931685	-0.03(-0.13,0.07)
Uzbekistan	0,897496132	0.90(0.62,1.18)
Vanuatu	-1,24563615	-1.25(-1.37,-1.12)
Venezuela (Bolivarian Republic of)	-1,822365694	-1.82(-1.88,-1.77)
Viet Nam	-1,287238097	-1.29(-1.46,-1.12)
Yemen	-0,305693576	-0.31(-0.34,-0.27)
Zambia	-1,049047363	-1.05(-1.10,-0.99)
Zimbabwe	-0,721412136	-0.72(-0.87,-0.57)

Table S2. Number of deaths from smoking-attributable dementia across 204 countries and territories in 2021.

age_name	location_name	cause_name	metric_name	2021-Death-Both
All ages	Afghanistan	Alzheimer's disease and other dementias	Number	28,78164899
All ages	Albania	Alzheimer's disease and other dementias	Number	57,56213783
All ages	Algeria	Alzheimer's disease and other dementias	Number	289,4946508
All ages	American Samoa	Alzheimer's disease and other dementias	Number	0,178196106
All ages	Andorra	Alzheimer's disease and other dementias	Number	1,42694056
All ages	Angola	Alzheimer's disease and other dementias	Number	27,35229949
All ages	Antigua and Barbuda	Alzheimer's disease and other dementias	Number	0,240208692
All ages	Argentina	Alzheimer's disease and other dementias	Number	255,4171851
All ages	Armenia	Alzheimer's disease and other dementias	Number	44,08579843
All ages	Australia	Alzheimer's disease and other dementias	Number	258,8702254
All ages	Austria	Alzheimer's disease and other dementias	Number	168,7507279
All ages	Azerbaijan	Alzheimer's disease and other dementias	Number	53,77596054
All ages	Bahamas	Alzheimer's disease and other dementias	Number	0,931095854
All ages	Bahrain	Alzheimer's disease and other dementias	Number	3,231027906
All ages	Bangladesh	Alzheimer's disease and other dementias	Number	781,1224363
All ages	Barbados	Alzheimer's disease and other dementias	Number	1,363792413
All ages	Belarus	Alzheimer's disease and other dementias	Number	90,88833229
All ages	Belgium	Alzheimer's disease and other dementias	Number	286,4724753
All ages	Belize	Alzheimer's disease and other dementias	Number	0,888283812
All ages	Benin	Alzheimer's disease and other dementias	Number	3,618207727
All ages	Bermuda	Alzheimer's disease and other dementias	Number	0,615221703
All ages	Bhutan	Alzheimer's disease and other dementias	Number	2,272537639
All ages	Bolivia (Plurinational State of)	Alzheimer's disease and other dementias	Number	16,90731879
All ages	Bosnia and Herzegovina	Alzheimer's disease and other dementias	Number	62,0572932
All ages	Botswana	Alzheimer's disease and other dementias	Number	5,066057628
All ages	Brazil	Alzheimer's disease and other dementias	Number	2152,528386
All ages	Brunei Darussalam	Alzheimer's disease and other dementias	Number	1,138559822
All ages	Bulgaria	Alzheimer's disease and other dementias	Number	54,4482708
All ages	Burkina Faso	Alzheimer's disease and other dementias	Number	6,324984948
All ages	Burundi	Alzheimer's disease and other dementias	Number	5,263596474
All ages	Cabo Verde	Alzheimer's disease and other dementias	Number	0,381804764
All ages	Cambodia	Alzheimer's disease and other dementias	Number	96,45846548
All ages	Cameroon	Alzheimer's disease and other dementias	Number	11,54633605
All ages	Canada	Alzheimer's disease and other dementias	Number	682,5848441
All ages	Central African Republic	Alzheimer's disease and other dementias	Number	2,217737613
All ages	Chad	Alzheimer's disease and other dementias	Number	7,038427406
All ages	Chile	Alzheimer's disease and other dementias	Number	65,3192321
All ages	China	Alzheimer's disease and other dementias	Number	24897,31037
All ages	Colombia	Alzheimer's disease and other dementias	Number	142,7166691
All ages	Comoros	Alzheimer's disease and other dementias	Number	1,457771164
All ages	Congo	Alzheimer's disease and other dementias	Number	6,323442236
All ages	Cook Islands	Alzheimer's disease and other dementias	Number	0,116777517
All ages	Costa Rica	Alzheimer's disease and other dementias	Number	23,93772213
All ages	Croatia	Alzheimer's disease and other dementias	Number	101,9879561
All ages	Cuba	Alzheimer's disease and other dementias	Number	106,7321132
All ages	Cyprus	Alzheimer's disease and other dementias	Number	17,4810456
All ages	Czechia	Alzheimer's disease and other dementias	Number	163,4118146
All ages	Côte d'Ivoire	Alzheimer's disease and other dementias	Number	12,35138972
All ages	Democratic People's Republic of Korea	Alzheimer's disease and other dementias	Number	139,8733606
All ages	Democratic Republic of the Congo	Alzheimer's disease and other dementias	Number	33,92008115
All ages	Denmark	Alzheimer's disease and other dementias	Number	197,4295606
All ages	Djibouti	Alzheimer's disease and other dementias	Number	2,010357711
All ages	Dominica	Alzheimer's disease and other dementias	Number	0,15931446
All ages	Dominican Republic	Alzheimer's disease and other dementias	Number	73,4675336
All ages	Ecuador	Alzheimer's disease and other dementias	Number	31,35479425
All ages	Egypt	Alzheimer's disease and other dementias	Number	395,4041067
All ages	El Salvador	Alzheimer's disease and other dementias	Number	14,71065852
All ages	Equatorial Guinea	Alzheimer's disease and other dementias	Number	0,803033093
All ages	Eritrea	Alzheimer's disease and other dementias	Number	1,783284544
All ages	Estonia	Alzheimer's disease and other dementias	Number	15,78760107
All ages	Eswatini	Alzheimer's disease and other dementias	Number	0,985668222
All ages	Ethiopia	Alzheimer's disease and other dementias	Number	43,30547789
All ages	Fiji	Alzheimer's disease and other dementias	Number	1,877990859
All ages	Finland	Alzheimer's disease and other dementias	Number	85,37596947
All ages	France	Alzheimer's disease and other dementias	Number	1100,954804
All ages	Gabon	Alzheimer's disease and other dementias	Number	1,725664915
All ages	Gambia	Alzheimer's disease and other dementias	Number	1,207957805
All ages	Georgia	Alzheimer's disease and other dementias	Number	47,01502665
All ages	Germany	Alzheimer's disease and other dementias	Number	2199,395806
All ages	Ghana	Alzheimer's disease and other dementias	Number	20,93877757
All ages	Greece	Alzheimer's disease and other dementias	Number	382,75832
All ages	Greenland	Alzheimer's disease and other dementias	Number	0,521168895
All ages	Grenada	Alzheimer's disease and other dementias	Number	0,158454934
All ages	Guam	Alzheimer's disease and other dementias	Number	0,798636999
All ages	Guatemala	Alzheimer's disease and other dementias	Number	27,99486076
All ages	Guinea	Alzheimer's disease and other dementias	Number	8,365239692
All ages	Guinea-Bissau	Alzheimer's disease and other dementias	Number	0,445273887
All ages	Guyana	Alzheimer's disease and other dementias	Number	1,10476452
All ages	Haiti	Alzheimer's disease and other dementias	Number	7,337254637
All ages	Honduras	Alzheimer's disease and other dementias	Number	24,83375667
All ages	Hungary	Alzheimer's disease and other dementias	Number	95,66390271
All ages	Iceland	Alzheimer's disease and other dementias	Number	6,110030339
All ages	India	Alzheimer's disease and other dementias	Number	4320,841898
All ages	Indonesia	Alzheimer's disease and other dementias	Number	1155,866383
All ages	Iran (Islamic Republic of)	Alzheimer's disease and other dementias	Number	399,8493626
All ages	Iraq	Alzheimer's disease and other dementias	Number	176,3906503
All ages	Ireland	Alzheimer's disease and other dementias	Number	83,93701759
All ages	Israel	Alzheimer's disease and other dementias	Number	96,59461727
All ages	Italy	Alzheimer's disease and other dementias	Number	1499,653523
All ages	Jamaica	Alzheimer's disease and other dementias	Number	12,25101097
All ages	Japan	Alzheimer's disease and other dementias	Number	3727,868944
All ages	Jordan	Alzheimer's disease and other dementias	Number	56,54184558
All ages	Kazakhstan	Alzheimer's disease and other dementias	Number	39,27212816
All ages	Kenya	Alzheimer's disease and other dementias	Number	39,21381733
All ages	Kiribati	Alzheimer's disease and other dementias	Number	0,499393257
All ages	Kuwait	Alzheimer's disease and other dementias	Number	20,94084974
All ages	Kyrgyzstan	Alzheimer's disease and other dementias	Number	28,38512055
All ages	Lao People's Democratic Republic	Alzheimer's disease and other dementias	Number	26,14821252
All ages	Latvia	Alzheimer's disease and other dementias	Number	16,88237721
All ages	Lebanon	Alzheimer's disease and other dementias	Number	108,2557837
All ages	Lesotho	Alzheimer's disease and other dementias	Number	4,687849299
All ages	Liberia	Alzheimer's disease and other dementias	Number	1,534746119
All ages	Libya	Alzheimer's disease and other dementias	Number	32,28643099
All ages	Lithuania	Alzheimer's disease and other dementias	Number	30,40600791
All ages	Luxembourg	Alzheimer's disease and other dementias	Number	6,75194739
All ages	Madagascar	Alzheimer's disease and other dementias	Number	8,642065571
All ages	Malawi	Alzheimer's disease and other dementias	Number	21,64537697
All ages	Malaysia	Alzheimer's disease and other dementias	Number	156,3852071
All ages	Maldives	Alzheimer's disease and other dementias	Number	2,552612882
All ages	Mali	Alzheimer's disease and other dementias	Number	17,36982166
All ages	Malta	Alzheimer's disease and other dementias	Number	6,208768675
All ages	Marshall Islands	Alzheimer's disease and other dementias	Number	0,084065779
All ages	Mauritania	Alzheimer's disease and other dementias	Number	2,161453227
All ages	Mauritius	Alzheimer's disease and other dementias	Number	10,30810009
All ages	Mexico	Alzheimer's disease and other dementias	Number	287,2316016
All ages	Micronesia (Federated States of)	Alzheimer's disease and other dementias	Number	0,313765141
All ages	Monaco	Alzheimer's disease and other dementias	Number	1,110063936
All ages	Mongolia	Alzheimer's disease and other dementias	Number	8,741161541
All ages	Montenegro	Alzheimer's disease and other dementias	Number	6,652448523
All ages	Morocco	Alzheimer's disease and other dementias	Number	89,16320157
All ages	Mozambique	Alzheimer's disease and other dementias	Number	21,17341084
All ages	Myanmar	Alzheimer's disease and other dementias	Number	239,8519392
All ages	Namibia	Alzheimer's disease and other dementias	Number	6,317717174
All ages	Nauru	Alzheimer's disease and other dementias	Number	0,019995694
All ages	Nepal	Alzheimer's disease and other dementias	Number	149,5635838
All ages	Netherlands	Alzheimer's disease and other dementias	Number	486,2339953
All ages	New Zealand	Alzheimer's disease and other dementias	Number	78,29161162
All ages	Nicaragua	Alzheimer's disease and other dementias	Number	14,1012972
All ages	Niger	Alzheimer's disease and other dementias	Number	6,307636383
All ages	Nigeria	Alzheimer's disease and other dementias	Number	58,41216337
All ages	Niue	Alzheimer's disease and other dementias	Number	0,008605282
All ages	North Macedonia	Alzheimer's disease and other dementias	Number	20,35605536
All ages	Northern Mariana Islands	Alzheimer's disease and other dementias	Number	0,166110887
All ages	Norway	Alzheimer's disease and other dementias	Number	79,15681802
All ages	Oman	Alzheimer's disease and other dementias	Number	4,239006345
All ages	Pakistan	Alzheimer's disease and other dementias	Number	409,7698713
All ages	Palau	Alzheimer's disease and other dementias	Number	0,06459877
All ages	Palestine	Alzheimer's disease and other dementias	Number	14,27530849
All ages	Panama	Alzheimer's disease and other dementias	Number	16,88585904
All ages	Papua New Guinea	Alzheimer's disease and other dementias	Number	11,35824263
All ages	Paraguay	Alzheimer's disease and other dementias	Number	67,17739144
All ages	Peru	Alzheimer's disease and other dementias	Number	92,9473746
All ages	Philippines	Alzheimer's disease and other dementias	Number	428,9312464
All ages	Poland	Alzheimer's disease and other dementias	Number	446,2248453
All ages	Portugal	Alzheimer's disease and other dementias	Number	90,42862916
All ages	Puerto Rico	Alzheimer's disease and other dementias	Number	34,98771683
All ages	Qatar	Alzheimer's disease and other dementias	Number	1,989149276
All ages	Republic of Korea	Alzheimer's disease and other dementias	Number	985,5230455
All ages	Republic of Moldova	Alzheimer's disease and other dementias	Number	26,58610239
All ages	Romania	Alzheimer's disease and other dementias	Number	151,0382086
All ages	Russian Federation	Alzheimer's disease and other dementias	Number	1131,324436
All ages	Rwanda	Alzheimer's disease and other dementias	Number	47,63613113
All ages	Saint Kitts and Nevis	Alzheimer's disease and other dementias	Number	0,092763259
All ages	Saint Lucia	Alzheimer's disease and other dementias	Number	0,552863267
All ages	Saint Vincent and the Grenadines	Alzheimer's disease and other dementias	Number	0,313059105
All ages	Samoa	Alzheimer's disease and other dementias	Number	0,860877173
All ages	San Marino	Alzheimer's disease and other dementias	Number	0,778982684
All ages	Sao Tome and Principe	Alzheimer's disease and other dementias	Number	0,071530341
All ages	Saudi Arabia	Alzheimer's disease and other dementias	Number	39,63636949
All ages	Senegal	Alzheimer's disease and other dementias	Number	7,639771746
All ages	Serbia	Alzheimer's disease and other dementias	Number	99,73478756
All ages	Seychelles	Alzheimer's disease and other dementias	Number	0,622168158
All ages	Sierra Leone	Alzheimer's disease and other dementias	Number	3,495190177
All ages	Singapore	Alzheimer's disease and other dementias	Number	23,2164194
All ages	Slovakia	Alzheimer's disease and other dementias	Number	46,18420313
All ages	Slovenia	Alzheimer's disease and other dementias	Number	24,63731032
All ages	Solomon Islands	Alzheimer's disease and other dementias	Number	1,514949
All ages	Somalia	Alzheimer's disease and other dementias	Number	7,430309174
All ages	South Africa	Alzheimer's disease and other dementias	Number	109,023217
All ages	South Sudan	Alzheimer's disease and other dementias	Number	7,323417088
All ages	Spain	Alzheimer's disease and other dementias	Number	695,7913017
All ages	Sri Lanka	Alzheimer's disease and other dementias	Number	70,99303673
All ages	Sudan	Alzheimer's disease and other dementias	Number	94,01589499
All ages	Suriname	Alzheimer's disease and other dementias	Number	1,997968876
All ages	Sweden	Alzheimer's disease and other dementias	Number	281,0144001
All ages	Switzerland	Alzheimer's disease and other dementias	Number	236,9530296
All ages	Syrian Arab Republic	Alzheimer's disease and other dementias	Number	73,54893473
All ages	Taiwan (Province of China)	Alzheimer's disease and other dementias	Number	296,2351173
All ages	Tajikistan	Alzheimer's disease and other dementias	Number	15,99613671
All ages	Thailand	Alzheimer's disease and other dementias	Number	743,0570958
All ages	Timor-Leste	Alzheimer's disease and other dementias	Number	4,214271309
All ages	Togo	Alzheimer's disease and other dementias	Number	5,803486118
All ages	Tokelau	Alzheimer's disease and other dementias	Number	0,007946792
All ages	Tonga	Alzheimer's disease and other dementias	Number	0,692625675
All ages	Trinidad and Tobago	Alzheimer's disease and other dementias	Number	5,370389917
All ages	Tunisia	Alzheimer's disease and other dementias	Number	132,4067672
All ages	Turkey	Alzheimer's disease and other dementias	Number	605,7450821
All ages	Turkmenistan	Alzheimer's disease and other dementias	Number	10,3066486
All ages	Tuvalu	Alzheimer's disease and other dementias	Number	0,046528796
All ages	Uganda	Alzheimer's disease and other dementias	Number	28,8996463
All ages	Ukraine	Alzheimer's disease and other dementias	Number	335,5645364
All ages	United Arab Emirates	Alzheimer's disease and other dementias	Number	9,338407573
All ages	United Kingdom	Alzheimer's disease and other dementias	Number	1425,15384
All ages	United Republic of Tanzania	Alzheimer's disease and other dementias	Number	86,16333752
All ages	United States of America	Alzheimer's disease and other dementias	Number	7091,073926
All ages	United States Virgin Islands	Alzheimer's disease and other dementias	Number	0,516080685
All ages	Uruguay	Alzheimer's disease and other dementias	Number	30,0436691
All ages	Uzbekistan	Alzheimer's disease and other dementias	Number	52,39365374
All ages	Vanuatu	Alzheimer's disease and other dementias	Number	0,373497388
All ages	Venezuela (Bolivarian Republic of)	Alzheimer's disease and other dementias	Number	102,7700207
All ages	Viet Nam	Alzheimer's disease and other dementias	Number	601,2202848
All ages	Yemen	Alzheimer's disease and other dementias	Number	85,50942766
All ages	Zambia	Alzheimer's disease and other dementias	Number	18,02923463
All ages	Zimbabwe	Alzheimer's disease and other dementias	Number	25,00014407

Table S3. Frontier analysis results for age-standardised DALY rates (ASDR) attributable to smoking-related dementias across 204 countries and territories in 2021.

location_name	SDI	year	ASDR-val	ASDR-frontier	effective difference range
Somalia	0,077688109	2021	17,31415732	17,31415732	0
Niger	0,168072774	2021	8,720112132	7,481412963	1,238699169
Chad	0,240436019	2021	12,76266357	6,142634913	6,620028659
Mali	0,268579941	2021	21,04478707	5,714777654	15,33000942
South Sudan	0,278371125	2021	20,78684298	5,710619756	15,07622323
Burkina Faso	0,285118402	2021	6,892614086	5,7121655	1,180448586
Burundi	0,289374365	2021	12,05379739	5,713346875	6,340450512
Central African Republic	0,30916769	2021	14,26185782	5,71110621	8,550751611
Mozambique	0,326462614	2021	21,02825449	5,711817804	15,31643669
Guinea	0,336401293	2021	13,5592261	5,714826309	7,844399791
Afghanistan	0,337199998	2021	32,07597931	5,714423198	26,36155611
Liberia	0,352442452	2021	7,81755992	5,712510366	2,105049554
Guinea-Bissau	0,353109621	2021	7,366086571	5,712583045	1,653503526
Sierra Leone	0,358665881	2021	9,874416829	5,714559322	4,159857507
Ethiopia	0,358823295	2021	9,244747838	5,710027194	3,534720644
Benin	0,373486574	2021	7,091436639	5,712397432	1,379039206
Democratic Republic of the Congo	0,383179849	2021	10,41094836	5,711724307	4,699224053
Malawi	0,384553634	2021	30,77043252	5,714937428	25,05549509
Madagascar	0,400246943	2021	10,81316416	5,713113596	5,100050563
Eritrea	0,403863943	2021	7,933812711	5,711525608	2,222287103
Senegal	0,408054193	2021	9,365654379	5,711344434	3,654309945
Togo	0,408533695	2021	17,3629835	5,711537357	11,65144614
Gambia	0,40971416	2021	11,82773247	5,71629323	6,111439242
Papua New Guinea	0,417797443	2021	28,31312287	5,712869875	22,60025299
Uganda	0,423261181	2021	19,51841135	5,711547137	13,80686421
C?te d'Ivoire	0,425941883	2021	12,17824376	5,711143915	6,467099843
Solomon Islands	0,429360316	2021	51,77961536	5,710560685	46,06905467
Nepal	0,433174635	2021	56,32987352	5,713532819	50,6163407
Rwanda	0,435588706	2021	78,46148997	5,714396852	72,74709312
Timor-Leste	0,444667619	2021	49,83654862	5,713724736	44,12282388
United Republic of Tanzania	0,446568273	2021	31,15089058	5,715751669	25,43513891
Haiti	0,448278285	2021	11,7659874	5,713981921	6,052005476
Yemen	0,450376375	2021	65,13229316	5,711816198	59,42047696
Angola	0,453721949	2021	27,7179322	5,710399503	22,0075327
Bhutan	0,473062378	2021	25,35140754	5,712077329	19,63933021
Vanuatu	0,473100706	2021	24,35876501	5,712915181	18,64584983
Cambodia	0,473621491	2021	77,84702867	5,710728163	72,13630051
Zimbabwe	0,473819486	2021	45,06532734	5,716541699	39,34878564
Comoros	0,475978688	2021	25,78415331	5,710978894	20,07317441
Cameroon	0,479691223	2021	9,737351424	5,715988393	4,021363032
Djibouti	0,487958371	2021	39,9195175	5,711138708	34,2083788
Lao People's Democratic Republic	0,489136091	2021	59,90683932	5,717614139	54,18922518
Bangladesh	0,492420885	2021	45,68869494	5,71279374	39,9759012
Mauritania	0,4989451	2021	9,155471796	5,712846965	3,442624832
Nigeria	0,503390833	2021	6,105714851	5,714304497	0,391410354
Pakistan	0,504028689	2021	33,31884503	5,715368296	27,60347673
Sao Tome and Principe	0,505413747	2021	6,491102456	5,717445631	0,773656825
Zambia	0,505948954	2021	29,99952573	5,711469219	24,28805651
Lesotho	0,510393066	2021	49,89217222	5,713953572	44,17821865
Honduras	0,513037248	2021	36,25954211	5,710823403	30,54871871
Kenya	0,523768077	2021	18,0937149	5,715327747	12,37838715
Nicaragua	0,523958472	2021	24,89721772	5,712185832	19,18503189
Kiribati	0,527186583	2021	84,26431738	5,712163936	78,55215344
Cabo Verde	0,533534539	2021	6,836627802	5,717350914	1,119276887
Myanmar	0,53390084	2021	44,60746426	5,711500391	38,89596387
Guatemala	0,539972424	2021	20,88932183	5,713225738	15,17609609
Tajikistan	0,541511187	2021	28,59498735	5,71182547	22,88316188
Sudan	0,541949735	2021	46,08340132	5,71508971	40,36831161
Morocco	0,562698301	2021	23,66447892	5,711608909	17,95287001
El Salvador	0,563775188	2021	16,99152964	5,713736476	11,27779317
Ghana	0,56493039	2021	13,03287651	5,710132586	7,322743927
Democratic People's Republic of Korea	0,569854634	2021	36,33343361	5,710905201	30,62252841
Marshall Islands	0,574091128	2021	31,95347259	5,715173949	26,23829864
India	0,575401649	2021	30,62391727	5,714669507	24,90924776
Tuvalu	0,576620529	2021	42,60809837	5,714014367	36,894084
Congo	0,583075236	2021	26,887382	5,71574581	21,17163619
Eswatini	0,585459713	2021	21,87266722	5,713571391	16,15909583
Micronesia (Federated States of)	0,587534967	2021	47,35079921	5,71231849	41,63848072
Samoa	0,593392769	2021	53,24059629	5,715252869	47,52534342
Venezuela (Bolivarian Republic of)	0,596513059	2021	27,15400136	5,714566468	21,43943489
Bolivia (Plurinational State of)	0,599010799	2021	16,96212036	5,711730569	11,25038979
Kyrgyzstan	0,603979328	2021	55,86031999	5,71124652	50,14907347
Egypt	0,606787094	2021	80,86982387	5,711982428	75,15784144
Belize	0,610229002	2021	25,58295311	5,711815375	19,87113774
Namibia	0,617564872	2021	46,84110546	5,714090654	41,1270148
Mongolia	0,617621565	2021	43,98625551	5,713160211	38,2730953
Dominican Republic	0,619388201	2021	50,30971231	5,710306207	44,5994061
Syrian Arab Republic	0,623004075	2021	60,30951525	5,71281345	54,5967018
Nauru	0,625177834	2021	41,99890209	5,711687868	36,28721422
Tonga	0,626349936	2021	60,27130299	5,712957085	54,5583459
Viet Nam	0,627933721	2021	50,82539036	5,715340703	45,11004966
Palestine	0,631011665	2021	60,4727157	5,71184501	54,76087069
Suriname	0,633665739	2021	26,58643633	5,715730599	20,87070573
Gabon	0,634691393	2021	16,40376014	5,712061335	10,69169881
Paraguay	0,635718099	2021	75,98210079	5,711936431	70,27016436
Saint Vincent and the Grenadines	0,637195963	2021	18,30922411	5,713282656	12,59594146
Botswana	0,642721629	2021	36,56216001	5,712370294	30,84978971
Guyana	0,650812335	2021	17,17210662	5,714500795	11,45760582
Maldives	0,650886627	2021	64,86395795	5,711354102	59,15260384
Philippines	0,651219329	2021	50,28627295	5,712120068	44,57415288
Brazil	0,653043887	2021	61,86578657	5,711527269	56,1542593
Colombia	0,655442913	2021	18,46599834	5,711792466	12,75420587
Indonesia	0,656868336	2021	55,04042178	5,710586149	49,32983563
Equatorial Guinea	0,657857456	2021	17,21450698	5,715856051	11,49865092
Algeria	0,659500924	2021	77,82855286	5,712906254	72,11564661
Ecuador	0,661017053	2021	14,91343775	5,710953795	9,202483956
Peru	0,662054037	2021	17,58002043	5,711580377	11,86844006
Uzbekistan	0,662621694	2021	21,00051497	5,712948017	15,28756695
Iraq	0,662626231	2021	75,77138359	5,711724368	70,05965922
Mexico	0,664575304	2021	18,15320891	5,711331425	12,44187748
Cuba	0,668729864	2021	38,14062357	5,713051563	32,42757201
Grenada	0,668993028	2021	13,8221998	5,717409189	8,104790616
Saint Lucia	0,672509735	2021	17,92969322	5,713556943	12,21613628
Fiji	0,675051631	2021	27,23746608	5,714783521	21,52268256
South Africa	0,679626598	2021	22,11857067	5,71314056	16,40543011
Turkmenistan	0,682160776	2021	25,11665352	5,715985558	19,40066796
Tunisia	0,682432216	2021	74,70817967	5,711942228	68,99623744
Thailand	0,682547933	2021	42,00117533	5,711845847	36,28932948
Jamaica	0,683263064	2021	26,48282184	5,713320469	20,76950137
Tokelau	0,686425621	2021	37,88443815	5,712417416	32,17202073
Azerbaijan	0,694851274	2021	51,73524454	5,711370322	46,02387422
Iran (Islamic Republic of)	0,697207398	2021	42,48795162	5,710756754	36,77719486
Costa Rica	0,700340477	2021	29,57085958	5,715694037	23,85516554
Sri Lanka	0,701534935	2021	21,74099869	5,711362118	16,02963657
Armenia	0,701833194	2021	63,82852468	5,713722688	58,114802
Albania	0,706849791	2021	91,2313806	5,710222098	85,5211585
Panama	0,708864828	2021	24,08374419	5,71272093	18,37102326
Turkey	0,712692673	2021	51,79645092	5,711657869	46,08479305
Mauritius	0,718260446	2021	42,68744278	5,712642372	36,97480041
Uruguay	0,719283445	2021	36,27817129	5,711112666	30,56705863
China	0,72162976	2021	91,49647848	5,713121859	85,78335662
Bosnia and Herzegovina	0,723077893	2021	67,2862394	5,714173808	61,57206559
Argentina	0,723122973	2021	35,49989881	5,7139894	29,78590941
American Samoa	0,723727533	2021	36,46671003	5,71497097	30,75173906
Kazakhstan	0,725144495	2021	23,26888433	5,711601841	17,55728248
Jordan	0,725307227	2021	84,35627861	5,711836827	78,64444178
Libya	0,725771399	2021	52,91100427	5,717277341	47,19372692
Niue	0,72622205	2021	31,46456642	5,713868227	25,75069819
Seychelles	0,730150775	2021	45,21182521	5,714269538	39,49755568
Republic of Moldova	0,732214875	2021	35,44349809	5,715068306	29,72842979
Georgia	0,732473604	2021	49,2062256	5,710664093	43,4955615
Malaysia	0,742523828	2021	45,66589623	5,71112451	39,95477172
Portugal	0,744151851	2021	23,64968712	5,715596584	17,93409053
Lebanon	0,744746351	2021	106,4404824	5,716338611	100,7241438
Barbados	0,746748764	2021	16,8484769	5,712445906	11,13603099
Dominica	0,746967185	2021	16,04149995	5,711579774	10,32992018
Antigua and Barbuda	0,749886887	2021	20,21384806	5,712629677	14,50121838
North Macedonia	0,750629703	2021	62,62577207	5,714203117	56,91156895
Bahrain	0,753043204	2021	54,75357477	5,711133945	49,04244083
Palau	0,754046931	2021	32,1330694	5,715415326	26,41765407
Saint Kitts and Nevis	0,754987055	2021	13,49218299	5,712729118	7,779453874
Ukraine	0,760773913	2021	31,0275025	5,71650757	25,31099493
Bulgaria	0,768150939	2021	34,48264364	5,715811238	28,7668324
Romania	0,768453864	2021	30,67639223	5,71397156	24,96242067
Trinidad and Tobago	0,768763254	2021	22,55923862	5,713534544	16,84570407
Spain	0,769283698	2021	37,69052775	5,714295699	31,97623205
Chile	0,771514716	2021	21,5929358	5,711236726	15,88169908
Northern Mariana Islands	0,771535213	2021	34,75577118	5,711283238	29,04448794
Oman	0,773391602	2021	25,71870714	5,711482343	20,0072248
Cook Islands	0,779109955	2021	34,20911588	5,711766798	28,49734908
Belarus	0,784484711	2021	41,83796755	5,715251362	36,12271618
Hungary	0,790754768	2021	37,68659033	5,712633778	31,97395655
Greece	0,791854408	2021	72,42870776	5,714590822	66,71411694
Serbia	0,792416294	2021	47,13848194	5,712069789	41,42641216
Montenegro	0,795800584	2021	60,66025735	5,711502173	54,94875517
Croatia	0,798341027	2021	66,8425493	5,712985996	61,12956331
Malta	0,801585034	2021	39,2488608	5,713961775	33,53489902
Guam	0,803982203	2021	28,98837555	5,715627185	23,27274837
Bahamas	0,805020668	2021	20,49877474	5,712011858	14,78676288
Italy	0,805773534	2021	51,99030017	5,711945367	46,2783548
Russian Federation	0,808536005	2021	36,75954794	5,713312837	31,04623511
Israel	0,809011652	2021	43,54298114	5,711602577	37,83137857
Brunei Darussalam	0,810234367	2021	37,47312179	5,712276221	31,76084557
Slovakia	0,81061053	2021	37,32054935	5,713597532	31,60695182
Poland	0,812042809	2021	44,60767935	5,71047841	38,89720094
Saudi Arabia	0,815143493	2021	28,78370496	5,714701494	23,06900347
Bermuda	0,821365422	2021	28,98553152	5,713988911	23,2715426
United States Virgin Islands	0,821830853	2021	21,2827786	5,710709652	15,57206895
Puerto Rico	0,825525847	2021	27,24331311	5,712197349	21,53111576
Greenland	0,826210336	2021	80,44404567	5,712483157	74,73156251
Czechia	0,828450433	2021	52,48643125	5,711597157	46,77483409
Latvia	0,830663516	2021	31,20540211	5,711327329	25,49407478
Cyprus	0,835630545	2021	59,57782808	5,712685698	53,86514238
France	0,838364875	2021	39,78165565	5,712284266	34,06937138
Slovenia	0,842430731	2021	41,00582982	5,716844872	35,28898495
Australia	0,844252814	2021	33,23759215	5,711287296	27,52630485
Estonia	0,844917787	2021	40,39016414	5,711896517	34,67826762
Kuwait	0,846651055	2021	65,95163949	5,711808219	60,23983127
Qatar	0,846860584	2021	34,66550129	5,71282048	28,95268081
United Arab Emirates	0,849317734	2021	62,3920743	5,710809227	56,68126508
New Zealand	0,849442499	2021	52,43993596	5,712280413	46,72765555
Belgium	0,853654016	2021	61,27260585	5,711969977	55,56063587
Austria	0,853837004	2021	53,98057755	5,711772037	48,26880552
Singapore	0,856097766	2021	19,73103106	5,713476476	14,01755458
Lithuania	0,856484049	2021	32,72096059	5,71163502	27,00932557
United Kingdom	0,859000182	2021	54,31982156	5,713415598	48,60640596
Finland	0,859831368	2021	37,34517848	5,712263304	31,63291518
United States of America	0,862448354	2021	70,94463874	5,711519012	65,23311973
Andorra	0,869444113	2021	52,36888643	5,712557788	46,65632864
Japan	0,871241813	2021	45,92846596	5,715458848	40,21300711
Canada	0,87317068	2021	59,36087015	5,717719718	53,64315043
Ireland	0,87375385	2021	58,18930882	5,712982997	52,47632583
Taiwan (Province of China)	0,874747053	2021	36,16791611	5,711663284	30,45625283
Iceland	0,87636168	2021	56,99318409	5,712477482	51,28070661
Luxembourg	0,884428955	2021	38,86257044	5,710733459	33,15183698
Republic of Korea	0,886675267	2021	62,67156393	5,713839573	56,95772436
Sweden	0,886880299	2021	66,43440106	5,711745778	60,72265528
San Marino	0,888005474	2021	47,43979491	5,710932949	41,72886196
Netherlands	0,888464256	2021	71,81598022	5,715722598	66,10025762
Denmark	0,896424204	2021	78,82394869	5,712370085	73,1115786
Germany	0,902957091	2021	59,02841837	5,71302808	53,31539029
Monaco	0,908262831	2021	54,97013663	5,712064827	49,25807181
Norway	0,91613281	2021	41,43082987	5,713636326	35,71719354
Switzerland	0,933059111	2021	59,85085328	5,714911897	54,13594138

Supplementary Methods

This supplementary section provides additional methodological details to support the analyses described in the main text, including definitions of key epidemiological indicators and further information on data processing and statistical procedures.

1 Sociodemographic Index (SDI)

The SDI is a composite index used to evaluate the level of development across countries and regions. It is derived from the geometric mean of three metrics: total fertility rate, average educational attainment, and per capita income[1]. SDI values range from 0 to 1, with higher values reflecting greater socioeconomic development. Based on these thresholds, the Global Burden of Disease (GBD) framework classifies 204 countries and territories into five development levels: low SDI (0-0.4658), low-middle SDI (0.4658-0.6188), middle SDI (0.6188-0.7120), high-middle SDI (0.7120-0.8103), and high SDI (0.8103-1.0000). This stratification systematically captures disparities in socioeconomic conditions that influence health outcomes across populations.

2 Disability-adjusted life years (DALYs)

DALYs are a widely used indicator of overall disease burden or injury, calculated by combining years of life lost (YLLs) and years lived with disability (YLDs), thereby offering more comprehensive measure of health damages than any single metric.

3 Age-standardised rates (ASRs)

ASR was calculated using the formula below:

$$ASR = \frac{\sum_{i=1}^{n} a_i w_i}{\sum_{i=1}^{n} w_i}$$

Here, n denotes the aggregate number of age groups; i indicates the current age group's index; a_i shows the indicator value for that age group, w_i reflects the number of individuals in age group$i$ under the standard population structure.

4 The estimated annual percentage change (EAPC)

To quantify the long-term trends in ASMR and ASDR attributable to smoking-related dementia, the estimated annual percentage change (EAPC) was calculated using linear regression. The regression model was specified as $\ln(y) = \alpha + \beta X + \varepsilon$, where $\ln(y)$ represents the natural logarithm of the ASR, α is the intercept, β is the slope, X denotes the calendar year, and ε is the error term. EAPC and its 95% confidence interval (CI) were computed as $100 \times [\exp(\beta) - 1]$, indicating the annual percentage change. If both the EAPC value and the lower bound of its 95% CI were above 0, the ASR was considered to be increasing; if both were below 0, it was considered to be decreasing; otherwise, the ASR was considered stable. The 95% CI for EAPC was obtained from the linear regression model. We applied Spearman rank correlation to examine the relationships between ASRs and SDI, and between EAPC and both ASRs and SDI. The Spearman rank correlation coefficient (R) ranges from –1 to 1. Values approaching either end of this range indicate stronger associations, whereas those near zero suggest weak or no correlation.

4 Frontier analysis

Frontier methods was used to investigate the relative efficiency of countries and territories in achieving health outcomes, with a focus on the relationship between SDI

and health performance[2]. This analysis defined the efficiency gap as the difference between the observed health burden in each country and the theoretical minimum burden at its corresponding SDI level, thereby quantifying relative efficiency. A standardised efficiency score was calculated to evaluate how closely each country approached the frontier line. Scores closer to 1 represented higher relative efficiency. To further examine the influence of development level and regional context on efficiency variation, stratified comparisons were conducted by SDI quintile and geographic region. Frontier analysis provides a comprehensive assessment of national and regional health performance, helping to identify potential areas for improvement and inform more targeted health intervention strategies.

5 Health inequality analysis

To characterise the distribution of the smoking-attributable burden of AD and other dementias across SDI regions, we conducted a health inequality analysis using the slope index of inequality (SII) and the concentration index[3]. The SII based on regression analysis to quantify absolute inequality in health outcomes across the socioeconomic gradient, exemplified by the SDI. In contrast, the concentration index assesses the extent to which health outcomes are concentrated across the entire socioeconomic distribution, reflecting the degree of inequality in health resource allocation. The concentration index values closer to zero suggest more equitable distribution.

6 Decomposition analysis

The Das Gupta decomposition method was applied to identify the structural drivers

underlying differences in overall disease burden[4]. Unlike conventional regression models that estimate associations between variables, this method decomposes changes in dementia-related deaths and DALYs during 1990−2021 into three distinct contributors: population ageing, population growth, and age-specific rates. It offers a clearer understanding of the mechanisms contributing to trends in the burden of AD and other dementias.

7 Bayesian age-period-cohort (BAPC) analysis

Additionally, we adopted the BAPC model to estimate disease burden in 2035, implemented with the BAPC package in R and based on observed data from 1990 to 2021. By capturing the relationships among age, period, and cohort effects, it supports more accurate modelling of temporal trends and facilitates projections over the medium and long term. To account for randomness in future incidence, the model automatically incorporates Poisson noise during prediction[5].

References

1 Institute for Health Metrics and Evaluation. Global burden of disease study 2021 (GBD 2021): socio-demographic index (SDI) 1950–2021. 2024 [cited 2025 May 17]. Available from: https://ghdx.healthdata.org/record/global-burden-disease-study-2021-gbd-2021-socio-demographic-index-sdi-1950%E2%80%932021.

2 Bai Z, Han J, An J, Wang H, Du X, Yang Z, et al. The global, regional, and national patterns of change in the burden of congenital birth defects, 1990–2021: an analysis of the Global Burden of Disease Study 2021 and forecast to 2040. EClinicalMedicine 2024;77:102873. https://doi.org/10.1016/j.eclinm.2024.102873.

3 World Health Organization. Handbook on health inequality monitoring with a special focus on low- and middle-income countries. 2013 [cited 2025 May 18]. Available from: https://www.who.int/publications/i/item/9789241548632.

4 Gupta PD. Standardization and decomposition of rates from cross-classified data. Genus 1994;50:171–96.

5 Riebler A, Held L. Projecting the future burden of cancer: Bayesian age-period-cohort analysis with integrated nested Laplace approximations. Biom J 2017;59:531–49. https://doi.org/10.1002/bimj.201500263.

© 2026 Hu X. et al.
